# Supplementary material for: Maternal, paternal, and other caregivers’ stimulation in low- and- middle-income countries
Source: PLoS One. 2020 Jul 10;15(7):e0236107. doi: 10.1371/journal.pone.0236107 (PMC7351158; doi:10.1371/journal.pone.0236107)
Supplement: S1 Table — (DOCX) [file pone.0236107.s001.docx]

**S1 Table**. Sample characteristics

| Country | Region | Income | Source | Year | Age (months) | Female % | Urban % |
| --- | --- | --- | --- | --- | --- | --- | --- |
| Afghanistan | South Asia | Low | MICS4 | 2010 | 47.1 | 48.7 | 14.6 |
| Algeria | Middle East/North Africa | Upper-middle | MICS4 | 2012 | 47.2 | 48.6 | 62.6 |
| Argentina | Latin America/Caribbean | Upper-middle | MICS4 | 2011 | 47.0 | 48.0 | 100.0 |
| Bangladesh | South Asia | Low | MICS5 | 2012 | 47.5 | 48.2 | 20.1 |
| Belarus | Europe and Central Asia | Upper-middle | MICS4 | 2012 | 47.5 | 49.6 | 71.6 |
| Belize | Latin America/Caribbean | Upper-middle | MICS5 | 2015 | 47.7 | 48.6 | 37.4 |
| Benin | Sub-Saharan Africa | Low | MICS5 | 2014 | 47.4 | 50.8 | 43.5 |
| Bosnia and Herzegovina | Europe and Central Asia | Upper-middle | MICS4 | 2011 | 46.9 | 50.3 | 34.7 |
| Burundi | Sub-Saharan Africa | Low | DHS | 2016 | 47.8 | 49.6 | 8.5 |
| Cameroon | Sub-Saharan Africa | Lower-middle | MICS5 | 2014 | 46.7 | 50.2 | 41.5 |
| Central African Republic | Sub-Saharan Africa | Low | MICS4 | 2010 | 46.0 | 51.6 | 35.9 |
| Congo | Sub-Saharan Africa | Lower-middle | MICS5 | 2014 | 48.2 | 48.0 | 63.5 |
| Costa Rica | Latin America/Caribbean | Upper-middle | MICS4 | 2011 | 47.6 | 49.0 | 55.5 |
| DR Congo | Sub-Saharan Africa | Low | DHS | 2013 | 44.4 | 51.1 | 29.9 |
| Dominican Republic | Latin America/Caribbean | Upper-middle | MICS5 | 2014 | 48.2 | 49.5 | 75.3 |
| East Timor | East Asia/Pacific | Lower-middle | DHS | 2016 | 47.3 | 48.0 | 29.3 |
| El Salvador | Latin America/Caribbean | Lower-middle | MICS5 | 2014 | 48.1 | 49.6 | 56.6 |
| Gambia | Sub-Saharan Africa | Low | MICS4 | 2010 | 46.2 | 49.3 | 42.5 |
| Ghana | Sub-Saharan Africa | Lower-middle | MICS4 | 2011 | 47.0 | 49.2 | 43.4 |
| Guinea | Sub-Saharan Africa | Low | MICS5 | 2016 | 47.2 | 48.4 | 32.6 |
| Guinea-Bissau | Sub-Saharan Africa | Low | MICS5 | 2014 | 46.9 | 50.6 | 37.1 |
| Guyana | Latin America/Caribbean | Lower-middle | MICS5 | 2014 | 48.3 | 47.8 | 21.1 |
| Iraq | Middle East/North Africa | Upper-middle | MICS6 | 2018 | 46.9 | 48.3 | 67.5 |
| Ivory Coast | Sub-Saharan Africa | Lower-middle | MICS5 | 2016 | 46.8 | 48.9 | 37.7 |
| Jamaica | Latin America/Caribbean | Upper-middle | MICS4 | 2011 | 48.0 | 45.8 | 56.0 |
| Jordan | Middle East/North Africa | Upper-middle | DHS | 2012 | 45.8 | 49.3 | 83.0 |
| Kazakhstan | Europe and Central Asia | Upper-middle | MICS5 | 2015 | 47.4 | 50.1 | 48.5 |
| Kosovo | Europe and Central Asia | Lower-middle | MICS5 | 2013 | 48.0 | 47.4 | 36.8 |
| Kyrgyzstan | Europe and Central Asia | Lower-middle | MICS5 | 2014 | 47.4 | 48.8 | 27.2 |
| Lao PDR | East Asia/Pacific | Lower-middle | MICS6 | 2017 | 47.0 | 48.8 | 27.6 |
| Lebanon | Middle East/North Africa | Upper-middle | MICS4 | 2011 | 47.4 | 47.2 | 66.0 |
| Macedonia | Europe and Central Asia | Upper-middle | MICS4 | 2011 | 48.1 | 47.7 | 50.6 |
| Malawi | Sub-Saharan Africa | Low | MICS5 | 2013 | 47.3 | 49.4 | 11.4 |
| Maldives | South Asia | Upper-middle | DHS | 2016 | 47.2 | 48.1 | 34.6 |
| Mali | Sub-Saharan Africa | Low | MICS5 | 2015 | 46.3 | 48.3 | 19.2 |
| Mauritania | Sub-Saharan Africa | Lower-middle | MICS5 | 2015 | 46.7 | 51.0 | 42.5 |
| Mexico | Latin America/Caribbean | Upper-middle | MICS5 | 2015 | 48.7 | 54.9 | 75.0 |
| Moldova | Europe and Central Asia | Lower-middle | MICS4 | 2012 | 47.3 | 46.5 | 37.5 |
| Mongolia | East Asia/Pacific | Lower-middle | MICS5 | 2013 | 47.3 | 49.8 | 58.3 |
| Montenegro | Europe and Central Asia | Upper-middle | MICS5 | 2013 | 48.1 | 43.4 | 64.5 |
| Nepal | South Asia | Low | MICS5 | 2014 | 47.7 | 49.4 | 13.3 |
| Nigeria | Sub-Saharan Africa | Lower-middle | MICS5 | 2016 | 47.3 | 49.4 | 30.6 |
| Palestine | Middle East/North Africa | Low | MICS5 | 2014 | 47.6 | 48.4 | 75.5 |
| Panama | Latin America/Caribbean | Upper-middle | MICS5 | 2013 | 47.6 | 44.9 | 61.0 |
| Paraguay | Latin America/Caribbean | Lower-middle | MICS5 | 2016 | 48.1 | 49.1 | 59.3 |
| Rwanda | Sub-Saharan Africa | Low | DHS | 2014 | 44.0 | 49.4 | 16.2 |
| Sao Tome and Principe | Sub-Saharan Africa | Lower-middle | MICS5 | 2014 | 47.8 | 49.7 | 66.3 |
| Senegal | Sub-Saharan Africa | Lower-middle | DHS | 2017 | 47.2 | 47.7 | 37.8 |
| Serbia | Europe and Central Asia | Upper-middle | MICS5 | 2014 | 48.5 | 47.3 | 65.4 |
| Sierra Leone | Sub-Saharan Africa | Low | MICS6 | 2017 | 47.3 | 49.8 | 37.7 |
| St. Lucia | Latin America/Caribbean | Upper-middle | MICS4 | 2012 | 47.6 | 51.8 | 17.3 |
| Suriname | Latin America/Caribbean | Upper-middle | MICS4 | 2010 | 47.1 | 53.4 | 60.3 |
| Swaziland | Sub-Saharan Africa | Lower-middle | MICS5 | 2014 | 47.7 | 49.9 | 20.1 |
| Thailand | East Asia/Pacific | Upper-middle | MICS5 | 2015 | 47.3 | 49.2 | 41.4 |
| Togo | Sub-Saharan Africa | Low | DHS | 2013 | 44.5 | 50.5 | 36.4 |
| Tunisia | Middle East/North Africa | Upper-middle | MICS4 | 2011 | 46.8 | 47.7 | 63.4 |
| Turkmenistan | Europe and Central Asia | Upper-middle | MICS5 | 2015 | 47.7 | 48.2 | 34.0 |
| Uganda | Sub-Saharan Africa | Low | DHS | 2016 | 47.5 | 50.3 | 21.3 |
| Ukraine | Europe and Central Asia | Lower-middle | MICS4 | 2012 | 47.7 | 50.5 | 69.8 |
| Uruguay | Latin America/Caribbean | Upper-middle | MICS4 | 2012 | 47.5 | 45.0 | 92.3 |
| Vietnam | East Asia/Pacific | Lower-middle | MICS5 | 2013 | 48.2 | 50.5 | 29.4 |
| Zimbabwe | Sub-Saharan Africa | Low | MICS5 | 2014 | 47.2 | 50.7 | 24.6 |
