# Supplementary material for: Maternal, paternal, and other caregivers’ stimulation in low- and- middle-income countries
Source: PLoS One. 2020 Jul 10;15(7):e0236107. doi: 10.1371/journal.pone.0236107 (PMC7351158; doi:10.1371/journal.pone.0236107)
Supplement: S2 Table — (DOCX) [file pone.0236107.s002.docx]

**S2 Table**. Included vs. excluded LMICs characteristics

|  | LMICs sample (*N*=62) | Non-MICS LMICs sample (*N*=74) | *p*-value for two sample equal means test |
| --- | --- | --- | --- |
| 1. HDI | 0.63 | 0.62 | 0.87 |
| 2. Gini index | 0.46 | 0.44 | 0.06 |
| 3. Proportion urban | 0.47 | 0.50 | 0.35 |
| 4. Proportion unemployed | 0.10 | 0.10 | 0.75 |
| 5. Homicide rate (x 100.000) | 9.21 | 8.64 | 0.78 |
| 6. Literacy rate | 81.78 | 79.35 | 0.48 |
| 7. Gender inequality index | 0.47 | 0.46 | 0.62 |

*Notes*. LMICs=Low- and Middle-Income Countries HDI=Human Development Index
