# Supplementary material for: Maternal, paternal, and other caregivers’ stimulation in low- and- middle-income countries
Source: PLoS One. 2020 Jul 10;15(7):e0236107. doi: 10.1371/journal.pone.0236107 (PMC7351158; doi:10.1371/journal.pone.0236107)
Supplement: S3 Table — (DOCX) [file pone.0236107.s003.docx]

**S3 Table**. Association between the proportion of children exposed to high stimulation and country-level characteristics for 62 countries

|  | % high maternal stimulation | % high paternal stimulation | % high stimulation by other caregivers |
| --- | --- | --- | --- |
|  |  |  |  |
| HDI | 1.34** | 0.47+ | 0.39 |
|  | (0.46) | (0.25) | (0.35) |
| Gini index | 16.73 | 0.88 | 5.38 |
|  | (27.10) | (14.66) | (20.65) |
| % Urban | -0.10 | -0.01 | -0.16 |
|  | (0.13) | (0.07) | (0.10) |
| Unemployment rate | -0.06 | 0.10 | 0.21 |
|  | (0.26) | (0.14) | (0.20) |
| East Asia and the Pacific | -22.51** | 0.23 | 5.40 |
|  | (8.14) | (4.41) | (6.21) |
| Latin America & the Caribbean | -17.19** | -5.95* | 2.62 |
|  | (6.10) | (3.30) | (4.65) |
| Middle East & North Africa | -24.50** | -7.59* | -3.79 |
|  | (8.25) | (4.46) | (6.29) |
| South Asia | -7.19 | -1.50 | 4.61 |
|  | (9.42) | (5.10) | (7.18) |
| Sub-Saharan Africa | -17.37+ | -3.30 | 7.11 |
|  | (9.97) | (5.39) | (7.59) |
| Low-income | -10.85 | -3.24 | -2.28 |
|  | (10.67) | (5.78) | (8.13) |
| Lower-middle income | -11.88+ | -6.33+ | -3.64 |
|  | (6.66) | (3.60) | (5.08) |
| Constant | -23.85 | -11.94 | -1.16 |
|  | (37.83) | (20.47) | (28.82) |
|  |  |  |  |
| R-squared | 0.81 | 0.62 | 0.18 |

Note. Standard errors in parentheses. Omitted region: Europe and Central Asia. Omitted income group: upper-middle income.

** p<0.01, + p<0.1
