# Supplementary material for: Maternal, paternal, and other caregivers’ stimulation in low- and- middle-income countries
Source: PLoS One. 2020 Jul 10;15(7):e0236107. doi: 10.1371/journal.pone.0236107 (PMC7351158; doi:10.1371/journal.pone.0236107)
Supplement: S4 Table — (DOCX) [file pone.0236107.s004.docx]

**S4 Table**. Percentage of children exposed to high stimulation (four of more activities)

| Country | Maternal | Paternal | Other caregivers |
| --- | --- | --- | --- |
| Afghanistan | 4.5(3.9, 5.1) | 4.0(3.4, 4.5) | 14.7(13.8, 15.7) |
| Algeria | 38.8(37.1, 40.5) | 17.4(15.9, 18.8) | 22.6(21.2, 24.1) |
| Argentina | 70.2(67.8, 72.7) | 22.3(20.1, 24.5) | 17.6(15.6, 19.6) |
| Bangladesh | 40.8(39.4, 42.1) | 10.1(9.2, 11.0) | 25.1(24.0, 26.3) |
| Belarus | 85.3(82.8, 87.8) | 28.6(25.4, 31.8) | 15.0(12.3, 17.6) |
| Belize | 68.9(65.5, 72.3) | 23.9(20.9, 27.0) | 25.4(22.3, 28.6) |
| Benin | 13.5(12.4, 14.6) | 5.2(4.5, 6.0) | 6.5(5.6, 7.4) |
| Bosnia and Herzegovina | 86.3(83.6, 88.9) | 30.9(27.5, 34.2) | 32.4(28.6, 36.1) |
| Burundi | 15.5(14.7, 16.3) | 2.6(2.2, 3.0) | 30.6(29.6, 31.5) |
| Cameroon | 13.6(12.2, 15.1) | 3.8(3.0, 4.6) | 24.2(22.4, 26.1) |
| Central African Republic | 21.9(20.3, 23.5) | 8.6(7.5, 9.7) | 34.8(32.9, 36.6) |
| Congo, Dem. Rep. | 4.7(4.0, 5.4) | 1.4(0.9, 1.9) | 11.8(10.7, 12.9) |
| Congo, Rep. | 26.7(24.6, 28.9) | 6.3(5.3, 7.3) | 20.0(18.1, 21.9) |
| Costa Rica | 47.5(41.6, 53.3) | 11.7(8.5, 15.0) | 15.9(11.5, 20.4) |
| Dominican Republic | 31.8(30.3, 33.4) | 6.6(5.8, 7.4) | 16.5(15.2, 17.7) |
| East Timor | 19.7(18.0, 21.5) | 4.9(4.0, 5.7) | 4.5(3.6, 5.5) |
| El Salvador | 40.6(38.3, 42.9) | 8.0(6.8, 9.3) | 6.8(5.6, 7.9) |
| Gambia | 6.7(5.8, 7.7) | 0.9(0.5, 1.3) | 36.4(34.4, 38.4) |
| Ghana | 11.3(9.6, 13.1) | 3.4(2.4, 4.4) | 18.7(16.5, 20.8) |
| Guinea | 14.7(13.4, 16.1) | 4.3(3.5, 5.0) | 13.5(12.2, 14.8) |
| Guinea-Bissau | 3.0(2.1, 3.8) | 0.3(0.1, 0.6) | 18.1(16.5, 19.8) |
| Guyana | 56.6(53.7, 59.4) | 16.1(14.0, 18.2) | 33.4(30.7, 36.1) |
| Iraq | 23.5(21.6, 25.5) | 10.2(8.8, 11.7) | 15.3(13.4, 17.2) |
| Ivory Coast | 13.0(11.5, 14.4) | 4.9(4.1, 5.8) | 8.7(7.5, 9.9) |
| Jamaica | 61.0(56.3, 65.7) | 13.6(10.3, 16.8) | 38.2(33.7, 42.8) |
| Jordan | 66.7(64.3, 69.2) | 20.7(18.5, 22.9) | 7.8(6.5, 9.2) |
| Kazakhstan | 51.3(48.2, 54.5) | 6.7(5.1, 8.4) | 27.8(25.0, 30.5) |
| Kosovo | 43.7(39.8, 47.6) | 6.1(4.1, 8.2) | 14.8(12.0, 17.6) |
| Lao PDR | 20.0(18.6, 21.3) | 11.0(9.9, 12.0) | 15.2(14.0, 16.4) |
| Kyrgyzstan | 30.3(27.8, 32.9) | 2.9(1.9, 3.8) | 24.3(21.9, 26.7) |
| Lebanon | 53.1(49.4, 56.9) | 9.3(7.1, 11.5) | 18.2(15.3, 21.1) |
| Macedonia | 60.6(55.7, 65.5) | 19.0(14.9, 23.0) | 20.7(16.9, 24.4) |
| Malawi | 9.8(8.9, 10.7) | 3.1(2.5, 3.7) | 10.4(9.5, 11.3) |
| Maldives | 86.9(84.4, 89.4) | 23.2(19.7, 26.8) | 20.9(17.6, 24.2) |
| Mali | 19.2(18.1, 20.3) | 5.1(4.5, 5.8) | 24.0(22.8, 25.2) |
| Mauritania | 20.7(19.2, 22.2) | 4.7(4.0, 5.5) | 18.4(17.0, 19.8) |
| Mexico | 62.7(58.3, 67.1) | 14.6(11.8, 17.5) | 11.3(8.8, 13.7) |
| Moldova | 69.0(65.2, 72.8) | 13.2(10.6, 15.9) | 23.0(19.5, 26.4) |
| Mongolia | 28.8(26.8, 30.7) | 9.9(8.6, 11.1) | 14.3(12.8, 15.8) |
| Montenegro | 92.0(89.8, 94.2) | 45.5(40.8, 50.3) | 31.7(27.3, 36.1) |
| Nepal | 30.6(28.2, 33.1) | 10.1(8.6, 11.6) | 29.7(27.2, 32.1) |
| Nigeria | 28.3(27.2, 29.3) | 10.9(10.2, 11.6) | 31.1(30.0, 32.2) |
| Palestine | 55.4(53.7, 57.2) | 12.3(11.1, 13.4) | 14.9(13.7, 16.2) |
| Panama | 54.0(50.4, 57.7) | 14.1(11.2, 17.0) | 15.9(13.2, 18.6) |
| Paraguay | 43.8(40.8, 46.8) | 16.8(14.4, 19.2) | 17.7(15.3, 20.1) |
| Rwanda | 11.3(10.0, 12.5) | 2.1(1.6, 2.7) | 20.2(18.6, 21.7) |
| Senegal | 6.3(5.3, 7.2) | 0.8(0.5, 1.2) | 12.9(11.6, 14.1) |
| Serbia | 89.6(87.5, 91.8) | 36.6(31.3, 41.9) | 18.2(14.6, 21.9) |
| Sierra Leone | 17.6(16.3, 18.9) | 7.4(6.5, 8.2) | 3.2(2.6, 3.8) |
| St. Lucia | 70.9(62.1, 79.7) | 22.3(14.2, 30.3) | 41.2(31.6, 50.7) |
| Suriname | 40.1(36.9, 43.4) | 7.8(5.9, 9.7) | 23.5(20.7, 26.3) |
| Swaziland | 16.7(13.9, 19.5) | 1.8(0.9, 2.8) | 16.9(14.5, 19.4) |
| São Tomé and Principe | 16.0(13.1, 18.9) | 3.0(1.8, 4.3) | 29.9(26.4, 33.4) |
| Thailand | 63.6(60.8, 66.3) | 34.4(31.6, 37.3) | 52.7(49.8, 55.6) |
| Togo | 8.4(7.3, 9.6) | 2.7(2.0, 3.4) | 11.0(9.7, 12.3) |
| Tunisia | 50.5(47.1, 53.9) | 19.8(17.0, 22.6) | 13.4(11.1, 15.6) |
| Turkmenistan | 82.0(80.0, 84.1) | 15.1(13.2, 17.0) | 10.9(9.1, 12.6) |
| Uganda | 18.5(17.3, 19.7) | 2.8(2.2, 3.3) | 22.4(21.2, 23.6) |
| Ukraine | 85.2(82.7, 87.7) | 26.8(23.9, 29.7) | 24.8(22.1, 27.4) |
| Uruguay | 77.7(71.9, 83.5) | 30.0(22.7, 37.2) | 38.3(30.3, 46.2) |
| Vietnam | 45.8(42.6, 48.9) | 15.2(12.9, 17.5) | 26.4(23.5, 29.3) |
| Zimbabwe | 17.7(16.5, 19.0) | 2.6(2.1, 3.1) | 17.8(16.6, 19.1) |
