# Supplementary material for: Maternal, paternal, and other caregivers’ stimulation in low- and- middle-income countries
Source: PLoS One. 2020 Jul 10;15(7):e0236107. doi: 10.1371/journal.pone.0236107 (PMC7351158; doi:10.1371/journal.pone.0236107)
Supplement: S5 Table — (DOCX) [file pone.0236107.s005.docx]

**S5 Table**. Prevalence of maternal, paternal and other caregivers' stimulation by activity (percentage of people engaged in each activity)

|  |  |  |  | **Maternal Stimulation** | | | | | |  | **Paternal Stimulation** | | | | | |  | **Other caregivers' Stimulation** | | | | | |
| --- | --- | --- | --- | --- | --- | --- | --- | --- | --- | --- | --- | --- | --- | --- | --- | --- | --- | --- | --- | --- | --- | --- | --- |
| Country | Sample | Survey year | Source | Reading | Telling stories | Singing | Going out | Playing | Count-ing |  | Reading | Telling stories | Singing | Going out | Playing | Count-ing |  | Reading | Telling stories | Singing | Going out | Playing | Counting |
| Afghanistan | 6775 | 2010 | MICS4 | 8.11 | 47.63 | 45.10 | 12.63 | 8.63 | 15.50 |  | 13.54 | 14.48 | 6.91 | 42.82 | 12.27 | 21.37 |  | 15.62 | 23.09 | 24.13 | 45.03 | 74.86 | 22.15 |
| Algeria | 5561 | 2012 | MICS4 | 32.49 | 55.18 | 53.27 | 34.58 | 49.88 | 64.27 |  | 16.28 | 22.49 | 17.35 | 66.40 | 40.43 | 34.42 |  | 18.13 | 23.97 | 30.51 | 27.22 | 57.14 | 38.66 |
| Argentina | 3662 | 2011 | MICS4 | 64.52 | 62.21 | 69.52 | 73.05 | 81.96 | 69.66 |  | 24.41 | 22.69 | 22.70 | 39.94 | 45.57 | 27.81 |  | 23.16 | 21.37 | 23.74 | 18.07 | 31.48 | 22.23 |
| Bangladesh | 8796 | 2012 | MICS5 | 63.73 | 58.49 | 39.05 | 48.84 | 24.41 | 58.17 |  | 18.01 | 18.30 | 9.82 | 47.82 | 12.26 | 18.70 |  | 29.06 | 29.54 | 23.79 | 42.32 | 70.68 | 28.63 |
| Belarus | 1411 | 2012 | MICS4 | 89.10 | 82.66 | 73.54 | 81.67 | 84.85 | 84.60 |  | 37.11 | 26.96 | 18.57 | 57.58 | 52.66 | 36.63 |  | 19.32 | 16.68 | 14.44 | 20.21 | 22.58 | 20.05 |
| Belize | 1093 | 2015 | MICS5 | 69.08 | 66.90 | 72.41 | 77.38 | 73.07 | 71.71 |  | 27.59 | 25.69 | 25.58 | 33.76 | 29.01 | 27.30 |  | 28.78 | 26.14 | 27.02 | 29.66 | 38.45 | 31.46 |
| Benin | 4822 | 2014 | MICS5 | 9.44 | 18.93 | 37.50 | 29.59 | 30.67 | 9.01 |  | 6.09 | 10.10 | 9.53 | 18.34 | 17.00 | 6.55 |  | 7.27 | 8.22 | 13.61 | 17.30 | 42.18 | 8.38 |
| Bosnia and Herzegovina | 1031 | 2011 | MICS4 | 79.03 | 84.96 | 81.32 | 89.61 | 91.97 | 79.57 |  | 29.38 | 36.96 | 30.01 | 62.62 | 67.29 | 34.34 |  | 29.30 | 37.61 | 35.51 | 46.50 | 52.48 | 33.79 |
| Burundi | 10380 | 2016 | DHS | 4.99 | 60.75 | 43.39 | 49.11 | 20.29 | 7.18 |  | 2.05 | 27.12 | 8.02 | 14.84 | 8.03 | 3.09 |  | 8.25 | 52.29 | 47.41 | 39.31 | 65.73 | 16.51 |
| Cameroon | 2815 | 2014 | MICS5 | 7.52 | 23.88 | 35.46 | 28.06 | 26.58 | 14.62 |  | 3.43 | 9.49 | 8.99 | 11.22 | 11.82 | 5.99 |  | 10.64 | 26.21 | 38.01 | 44.55 | 64.51 | 22.76 |
| Central African Republic | 3771 | 2010 | MICS4 | 4.91 | 26.91 | 46.18 | 39.73 | 28.18 | 41.74 |  | 5.53 | 18.88 | 15.57 | 23.21 | 14.95 | 20.53 |  | 9.26 | 31.26 | 52.25 | 49.27 | 73.60 | 46.45 |
| Congo | 3608 | 2014 | MICS5 | 22.41 | 34.33 | 49.04 | 52.04 | 31.09 | 31.42 |  | 7.94 | 12.02 | 12.23 | 22.30 | 13.04 | 10.71 |  | 19.59 | 22.55 | 27.82 | 23.34 | 52.41 | 28.83 |
| Costa Rica | 906 | 2011 | MICS4 | 47.11 | 34.11 | 55.37 | 57.65 | 70.41 | 67.91 |  | 13.53 | 11.15 | 17.70 | 23.39 | 36.01 | 21.94 |  | 21.12 | 17.02 | 23.47 | 17.14 | 36.03 | 23.08 |
| Dominican Republic | 7862 | 2014 | MICS5 | 30.96 | 34.06 | 46.37 | 53.35 | 51.34 | 28.63 |  | 6.79 | 9.82 | 11.74 | 22.39 | 20.21 | 9.65 |  | 19.34 | 21.47 | 24.27 | 16.77 | 39.43 | 21.10 |
| DR Congo^(a)^ | 6607 | 2013 | DHS | 3.31 | 24.73 | 33.61 | 28.51 | 14.15 | 21.46 |  | 3.03 | 11.93 | 9.51 | 17.77 | 6.47 | 11.21 |  | 7.84 | 26.34 | 42.43 | 37.39 | 63.62 | 37.75 |
| East Timor | 2825 | 2016 | DHS | 61.96 | 63.93 | 74.10 | 65.93 | 65.21 | 54.17 |  | 19.01 | 20.41 | 17.97 | 31.71 | 25.31 | 19.22 |  | 16.61 | 16.48 | 13.27 | 12.98 | 31.61 | 20.55 |
| El Salvador | 2987 | 2014 | MICS5 | 35.75 | 31.79 | 52.89 | 54.52 | 60.22 | 48.89 |  | 11.60 | 9.98 | 13.69 | 21.01 | 24.56 | 11.70 |  | 14.04 | 10.67 | 13.00 | 8.82 | 19.18 | 10.07 |
| Gambia | 4023 | 2010 | MICS4 | 2.58 | 12.21 | 26.06 | 40.50 | 16.96 | 7.45 |  | 1.65 | 2.33 | 2.47 | 15.08 | 6.35 | 4.18 |  | 15.38 | 36.18 | 65.14 | 58.26 | 80.08 | 28.71 |
| Ghana | 3067 | 2011 | MICS4 | 11.37 | 11.99 | 26.80 | 32.32 | 41.85 | 15.83 |  | 6.88 | 5.85 | 6.57 | 13.75 | 18.08 | 7.75 |  | 18.38 | 15.76 | 25.44 | 33.28 | 54.23 | 26.39 |
| Guinea | 3150 | 2016 | MICS5 | 8.73 | 19.28 | 48.83 | 47.73 | 49.20 | 8.79 |  | 3.21 | 8.46 | 12.38 | 26.17 | 31.75 | 5.17 |  | 7.45 | 14.85 | 35.35 | 43.54 | 72.78 | 14.35 |
| Guinea-Bissau | 2943 | 2014 | MICS5 | 3.29 | 10.43 | 36.65 | 18.13 | 0.89 | 16.41 |  | 2.01 | 6.10 | 3.68 | 7.18 | 1.06 | 2.92 |  | 10.27 | 23.72 | 32.55 | 35.62 | 43.45 | 24.83 |
| Guyana | 1328 | 2014 | MICS5 | 57.84 | 56.03 | 56.86 | 56.55 | 61.32 | 66.88 |  | 19.28 | 19.66 | 15.23 | 27.29 | 32.08 | 21.44 |  | 34.45 | 33.17 | 33.41 | 39.34 | 50.62 | 37.96 |
| Iraq | 7014 | 2018 | MICS6 | 21.15 | 33.28 | 35.56 | 35.83 | 41.84 | 38.42 |  | 10.69 | 12.29 | 15.25 | 47.05 | 34.75 | 17.12 |  | 14.38 | 17.34 | 25.69 | 23.57 | 55.16 | 22.63 |
| Ivory Coast | 3680 | 2016 | MICS5 | 7.10 | 16.23 | 27.10 | 48.90 | 46.74 | 12.96 |  | 4.72 | 8.56 | 6.69 | 26.62 | 26.73 | 7.42 |  | 9.89 | 7.87 | 15.04 | 31.29 | 64.10 | 14.90 |
| Jamaica | 668 | 2011 | MICS4 | 67.18 | 55.96 | 60.26 | 66.33 | 69.82 | 68.42 |  | 14.96 | 12.29 | 12.10 | 19.04 | 23.47 | 17.91 |  | 41.84 | 34.88 | 39.29 | 37.33 | 48.40 | 39.97 |
| Jordan | 4134 | 2012 | DHS | 57.56 | 62.86 | 71.35 | 71.81 | 75.01 | 72.13 |  | 16.86 | 20.44 | 27.03 | 57.50 | 46.39 | 28.57 |  | 8.58 | 8.03 | 11.53 | 8.50 | 17.87 | 11.23 |
| Kazakhstan | 2245 | 2015 | MICS5 | 55.85 | 58.00 | 56.85 | 59.43 | 51.31 | 56.31 |  | 9.87 | 11.00 | 5.91 | 39.09 | 34.72 | 10.99 |  | 27.88 | 31.79 | 28.65 | 37.21 | 51.36 | 30.98 |
| Kosovo | 660 | 2013 | MICS5 | 38.52 | 49.51 | 49.56 | 55.87 | 60.43 | 52.20 |  | 8.39 | 14.13 | 7.67 | 33.17 | 27.02 | 13.12 |  | 15.72 | 21.93 | 19.08 | 27.13 | 39.53 | 18.43 |
| Kyrgyzstan | 1783 | 2014 | MICS5 | 42.14 | 52.73 | 38.74 | 37.94 | 25.10 | 46.55 |  | 5.10 | 9.20 | 6.27 | 25.02 | 14.70 | 14.71 |  | 22.67 | 29.82 | 30.49 | 38.44 | 44.95 | 28.38 |
| Lao PDR | 4761 | 2017 | MICS6 | 24.64 | 21.02 | 22.36 | 42.28 | 33.02 | 29.24 |  | 15.36 | 14.32 | 14.61 | 29.22 | 23.30 | 20.58 |  | 18.78 | 15.52 | 23.10 | 30.15 | 45.34 | 24.34 |
| Lebanon | 705 | 2011 | MICS4 | 44.83 | 58.22 | 56.34 | 82.40 | 51.02 | 64.84 |  | 8.24 | 16.51 | 11.24 | 49.05 | 31.62 | 20.13 |  | 20.67 | 25.05 | 29.53 | 12.92 | 56.72 | 25.01 |
| Macedonia | 558 | 2011 | MICS4 | 57.45 | 63.10 | 66.75 | 63.34 | 64.21 | 66.17 |  | 18.75 | 25.54 | 16.24 | 52.56 | 45.20 | 29.23 |  | 22.15 | 31.63 | 28.90 | 37.80 | 47.57 | 26.73 |
| Malawi | 7719 | 2013 | MICS5 | 8.67 | 13.41 | 28.57 | 36.64 | 29.37 | 10.50 |  | 5.15 | 6.58 | 6.39 | 12.29 | 9.45 | 5.98 |  | 9.16 | 10.77 | 16.63 | 18.68 | 27.19 | 12.11 |
| Maldives | 1285 | 2016 | DHS | 86.17 | 88.51 | 77.95 | 90.11 | 77.53 | 90.50 |  | 24.38 | 25.88 | 21.15 | 37.94 | 27.44 | 26.03 |  | 27.34 | 23.03 | 20.59 | 23.17 | 35.58 | 26.13 |
| Mali | 6481 | 2015 | MICS5 | 13.26 | 32.76 | 37.27 | 34.42 | 26.93 | 16.92 |  | 6.70 | 14.58 | 9.95 | 16.32 | 13.64 | 9.59 |  | 15.76 | 32.79 | 37.76 | 43.88 | 72.47 | 27.45 |
| Mauritania | 4418 | 2015 | MICS5 | 25.07 | 36.34 | 34.01 | 23.82 | 26.17 | 30.34 |  | 7.75 | 12.38 | 7.75 | 8.88 | 11.32 | 12.20 |  | 17.37 | 24.71 | 24.74 | 25.93 | 54.94 | 26.72 |
| Mexico | 3355 | 2015 | MICS5 | 55.61 | 51.27 | 65.25 | 68.66 | 75.93 | 68.99 |  | 16.48 | 17.62 | 22.39 | 29.41 | 30.03 | 17.94 |  | 15.33 | 15.64 | 16.80 | 11.82 | 22.03 | 14.05 |
| Moldova | 733 | 2012 | MICS4 | 60.21 | 65.68 | 57.20 | 77.15 | 77.57 | 71.99 |  | 15.18 | 16.54 | 8.13 | 32.84 | 38.19 | 18.95 |  | 24.31 | 26.02 | 20.39 | 28.73 | 36.65 | 24.06 |
| Mongolia | 2361 | 2013 | MICS5 | 31.00 | 29.07 | 46.36 | 38.32 | 38.60 | 45.37 |  | 13.76 | 11.92 | 14.35 | 24.05 | 28.92 | 19.01 |  | 19.73 | 17.82 | 16.52 | 16.01 | 28.85 | 22.82 |
| Montenegro | 645 | 2013 | MICS5 | 88.36 | 93.94 | 89.34 | 91.64 | 92.33 | 86.04 |  | 54.16 | 51.52 | 41.70 | 73.55 | 70.96 | 38.46 |  | 32.92 | 33.21 | 31.96 | 43.77 | 47.00 | 29.64 |
| Nepal | 2261 | 2014 | MICS5 | 36.50 | 43.82 | 41.95 | 50.90 | 31.27 | 31.33 |  | 19.33 | 17.08 | 12.28 | 31.74 | 12.98 | 15.45 |  | 29.91 | 30.33 | 35.05 | 42.58 | 62.63 | 33.89 |
| Nigeria | 11567 | 2016 | MICS5 | 29.03 | 38.95 | 40.97 | 40.66 | 41.96 | 25.93 |  | 14.89 | 17.39 | 13.71 | 25.18 | 26.45 | 13.72 |  | 24.93 | 33.24 | 36.75 | 51.99 | 72.87 | 32.94 |
| Palestine | 3220 | 2014 | MICS5 | 37.62 | 59.14 | 59.96 | 67.60 | 66.82 | 71.40 |  | 10.25 | 18.71 | 15.18 | 46.65 | 43.59 | 26.94 |  | 13.67 | 18.51 | 23.21 | 17.04 | 41.67 | 24.57 |
| Panama | 2315 | 2013 | MICS5 | 49.50 | 45.18 | 59.82 | 68.34 | 61.79 | 56.84 |  | 14.78 | 13.69 | 18.32 | 34.06 | 28.50 | 16.96 |  | 20.53 | 19.25 | 18.77 | 18.21 | 25.68 | 17.66 |
| Paraguay | 1835 | 2016 | MICS5 | 43.26 | 38.43 | 52.01 | 58.52 | 58.14 | 53.77 |  | 18.83 | 17.31 | 21.54 | 35.24 | 30.76 | 22.16 |  | 22.09 | 17.90 | 22.22 | 19.84 | 31.34 | 22.22 |
| Rwanda | 2868 | 2014 | DHS | 6.77 | 14.44 | 24.27 | 32.03 | 33.43 | 23.86 |  | 3.28 | 4.97 | 5.65 | 10.92 | 12.39 | 10.62 |  | 15.72 | 24.93 | 28.91 | 26.87 | 41.77 | 29.48 |
| Sao Tome and Principe | 852 | 2014 | MICS5 | 15.33 | 20.66 | 30.96 | 44.70 | 26.75 | 18.89 |  | 5.12 | 7.45 | 6.42 | 13.23 | 8.36 | 5.82 |  | 29.74 | 33.13 | 39.56 | 25.94 | 65.40 | 41.52 |
| Senegal | 4593 | 2017 | DHS | 4.49 | 14.45 | 25.99 | 20.03 | 21.65 | 7.66 |  | 1.59 | 3.71 | 4.62 | 7.62 | 6.16 | 2.24 |  | 10.75 | 17.91 | 21.75 | 29.45 | 62.01 | 14.39 |
| Serbia | 1191 | 2014 | MICS5 | 81.10 | 83.86 | 84.18 | 87.87 | 94.89 | 84.22 |  | 35.91 | 41.55 | 31.86 | 68.03 | 74.00 | 37.47 |  | 17.97 | 19.79 | 17.44 | 25.52 | 34.59 | 16.77 |
| Sierra Leone | 4737 | 2017 | MICS6 | 12.54 | 23.66 | 34.65 | 34.96 | 41.46 | 12.09 |  | 8.18 | 11.42 | 10.51 | 21.04 | 23.24 | 7.51 |  | 6.14 | 5.70 | 6.81 | 8.45 | 16.24 | 5.19 |
| St. Lucia | 122 | 2012 | MICS4 | 72.40 | 64.39 | 70.66 | 79.07 | 79.02 | 75.94 |  | 20.68 | 19.55 | 23.11 | 37.68 | 43.34 | 30.62 |  | 44.17 | 37.76 | 41.43 | 46.92 | 57.62 | 48.18 |
| Suriname | 1283 | 2010 | MICS4 | 31.65 | 36.42 | 52.78 | 50.11 | 49.33 | 57.85 |  | 6.46 | 8.23 | 10.27 | 16.89 | 16.37 | 14.20 |  | 22.92 | 25.39 | 30.97 | 29.54 | 44.28 | 30.27 |
| Swaziland | 1074 | 2014 | MICS5 | 13.11 | 14.90 | 26.78 | 31.75 | 24.23 | 26.74 |  | 3.28 | 2.28 | 2.83 | 8.30 | 6.99 | 5.54 |  | 13.68 | 18.02 | 24.21 | 28.65 | 32.90 | 23.65 |
| Thailand | 5534 | 2015 | MICS5 | 62.68 | 52.66 | 52.90 | 67.50 | 68.39 | 66.79 |  | 36.58 | 27.40 | 28.32 | 49.66 | 51.99 | 39.39 |  | 52.46 | 39.49 | 47.28 | 63.74 | 72.44 | 56.67 |
| Togo | 2512 | 2013 | DHS | 36.95 | 63.67 | 66.23 | 54.07 | 38.56 | 37.45 |  | 28.57 | 25.86 | 15.43 | 26.71 | 19.13 | 26.06 |  | 52.26 | 49.52 | 46.07 | 55.60 | 76.85 | 58.94 |
| Tunisia | 1162 | 2011 | MICS4 | 37.34 | 53.39 | 67.15 | 52.01 | 73.35 | 56.79 |  | 17.32 | 23.64 | 17.19 | 46.51 | 54.75 | 30.30 |  | 14.80 | 17.00 | 20.03 | 12.85 | 41.12 | 22.50 |
| Turkmenistan | 1497 | 2015 | MICS5 | 85.58 | 82.72 | 83.69 | 75.81 | 76.47 | 78.03 |  | 22.21 | 15.98 | 7.01 | 55.27 | 44.95 | 25.52 |  | 14.20 | 19.13 | 13.21 | 23.19 | 24.68 | 14.97 |
| Uganda | 6158 | 2016 | DHS | 19.45 | 30.40 | 40.47 | 32.80 | 33.45 | 26.18 |  | 5.22 | 7.50 | 7.53 | 8.34 | 10.18 | 7.29 |  | 20.34 | 25.82 | 36.57 | 32.68 | 49.65 | 29.41 |
| Ukraine | 1901 | 2012 | MICS4 | 90.33 | 85.05 | 73.93 | 84.18 | 86.49 | 85.39 |  | 34.50 | 27.08 | 9.33 | 59.61 | 62.38 | 37.33 |  | 28.23 | 29.49 | 23.67 | 36.30 | 39.32 | 28.74 |
| Uruguay | 747 | 2012 | MICS4 | 73.80 | 68.01 | 81.69 | 78.42 | 85.79 | 73.92 |  | 33.39 | 30.91 | 36.34 | 47.34 | 51.79 | 36.79 |  | 41.88 | 37.53 | 37.27 | 36.30 | 52.86 | 43.16 |
| Vietnam | 1185 | 2013 | MICS5 | 32.10 | 42.51 | 60.91 | 52.77 | 60.92 | 59.56 |  | 14.60 | 14.77 | 18.34 | 43.25 | 42.78 | 27.54 |  | 18.66 | 25.39 | 32.72 | 40.71 | 57.20 | 36.28 |
| Zimbabwe | 3907 | 2014 | MICS5 | 10.86 | 24.31 | 36.79 | 38.25 | 26.78 | 19.94 |  | 3.82 | 9.75 | 7.16 | 12.16 | 10.04 | 5.35 |  | 14.37 | 24.08 | 28.84 | 22.36 | 32.77 | 19.28 |
| Note. (a) In this |  |  |  |  |  |  |  |  |  |  |  |  |  |  |  |  |  |  |  |  |  |  |  |
