# Supplementary material for: Maternal, paternal, and other caregivers’ stimulation in low- and- middle-income countries
Source: PLoS One. 2020 Jul 10;15(7):e0236107. doi: 10.1371/journal.pone.0236107 (PMC7351158; doi:10.1371/journal.pone.0236107)
Supplement: S6 Table — (DOCX) [file pone.0236107.s006.docx]

**S6 Table**. Wealth disparities in the percentage of children exposed to high maternal stimulation

| Country | Richest | Poorest | Difference (richest - poorest) |
| --- | --- | --- | --- |
| Afghanistan | 7.0(5.5, 8.5) | 3.9(2.7, 5.1) | 3.1(1.1, 5.0) |
| Algeria | 48.7(45.1, 52.4) | 26.0(22.4, 29.6) | 22.7(17.6, 27.8) |
| Bangladesh | 58.4(55.4, 61.3) | 24.1(21.8, 26.4) | 34.3(30.5, 38.0) |
| Belarus | 83.9(78.6, 89.2) | 82.9(76.7, 89.1) | 1.0(-7.2, 9.2) |
| Belize | 79.1(72.3, 85.9) | 56.2(49.0, 63.5) | 22.8(12.9, 32.8) |
| Benin | 25.1(21.4, 28.7) | 11.5(9.2, 13.7) | 13.6(9.3, 17.8) |
| Bosnia and Herzegovina | 95.2(92.5, 98.0) | 72.8(66.4, 79.3) | 22.4(15.4, 29.4) |
| Burundi | 27.1(24.6, 29.6) | 13.8(12.2, 15.4) | 13.3(10.4, 16.2) |
| Cameroon | 20.9(17.0, 24.7) | 14.8(11.7, 18.0) | 6.1(1.1, 11.0) |
| Central African Republic | 26.8(23.1, 30.5) | 16.6(13.6, 19.6) | 10.2(5.4, 14.9) |
| Congo, Dem. Rep. | 8.1(6.1, 10.1) | 1.4(0.8, 2.1) | 6.6(4.5, 8.7) |
| Congo, Rep. | 34.0(30.0, 38.0) | 15.6(12.6, 18.6) | 18.4(13.4, 23.4) |
| Costa Rica | 66.6(56.3, 76.9) | 32.1(22.0, 42.2) | 34.5(20.0, 49.0) |
| Dominican Republic | 42.7(38.9, 46.5) | 14.0(11.7, 16.4) | 28.6(24.2, 33.1) |
| East Timor | 23.0(18.6, 27.5) | 16.6(13.3, 19.9) | 6.5(0.9, 12.0) |
| El Salvador | 57.0(51.3, 62.7) | 27.9(23.7, 32.0) | 29.2(22.1, 36.2) |
| Gambia | 11.0(8.2, 13.8) | 5.6(4.1, 7.0) | 5.4(2.2, 8.6) |
| Ghana | 21.6(17.5, 25.7) | 2.0(0.8, 3.2) | 19.5(15.2, 23.8) |
| Guinea | 21.0(17.5, 24.5) | 9.1(6.8, 11.4) | 12.0(7.8, 16.1) |
| Guinea-Bissau | 7.0(4.5, 9.4) | 0.7(0.3, 1.1) | 6.3(3.7, 8.8) |
| Guyana | 66.6(60.7, 72.5) | 50.6(44.0, 57.2) | 16.0(7.1, 24.9) |
| Ivory Coast | 23.9(20.2, 27.7) | 7.4(5.3, 9.6) | 16.5(12.2, 20.8) |
| Iraq | 28.9(25.1, 32.7) | 10.5(8.4, 12.7) | 18.4(14.0, 22.8) |
| Jamaica | 67.3(53.4, 81.3) | 50.3(40.5, 60.0) | 17.1(-0.0, 34.2) |
| Jordan | 72.9(67.9, 77.8) | 53.6(48.1, 59.1) | 19.3(11.9, 26.7) |
| Kazakhstan | 68.5(62.9, 74.1) | 37.5(31.4, 43.6) | 31.0(22.8, 39.3) |
| Kosovo | 65.0(56.7, 73.4) | 33.1(25.1, 41.1) | 31.9(20.3, 43.6) |
| Lao PDR | 37.9(34.5, 41.4) | 9.4(7.4, 11.4) | 28.5(24.5, 32.5) |
| Kyrgyzstan | 48.0(41.7, 54.4) | 17.8(13.5, 22.1) | 30.2(22.5, 37.9) |
| Lebanon | 67.6(59.9, 75.3) | 37.5(29.2, 45.8) | 30.1(18.7, 41.5) |
| Macedonia | 79.5(70.7, 88.4) | 47.4(34.7, 60.1) | 32.1(16.5, 47.7) |
| Malawi | 13.0(10.6, 15.3) | 7.4(5.7, 9.1) | 5.6(2.7, 8.4) |
| Maldives | 88.2(83.2, 93.2) | 83.7(78.9, 88.4) | 4.5(-2.4, 11.5) |
| Mali | 23.9(21.2, 26.6) | 19.1(16.6, 21.6) | 4.8(1.2, 8.5) |
| Mauritania | 35.9(31.6, 40.1) | 14.3(11.8, 16.7) | 21.6(16.7, 26.5) |
| Mexico | 77.6(68.4, 86.8) | 46.8(40.4, 53.1) | 30.8(19.6, 42.0) |
| Moldova | 82.1(75.5, 88.7) | 56.5(48.2, 64.8) | 25.6(15.0, 36.3) |
| Mongolia | 42.6(37.8, 47.4) | 20.4(16.7, 24.1) | 22.2(16.1, 28.2) |
| Montenegro | 94.7(90.2, 99.2) | 81.6(74.5, 88.7) | 13.1(4.7, 21.6) |
| Nepal | 48.0(42.8, 53.2) | 16.8(12.2, 21.3) | 31.2(24.4, 38.1) |
| Nigeria | 57.8(55.3, 60.4) | 10.8(9.4, 12.3) | 47.0(44.1, 49.9) |
| Palestine | 65.7(62.0, 69.4) | 47.0(43.0, 50.9) | 18.7(13.3, 24.1) |
| Panama | 68.5(62.2, 74.9) | 14.8(10.6, 19.0) | 53.7(46.1, 61.3) |
| Paraguay | 70.4(64.6, 76.1) | 20.4(14.8, 26.1) | 50.0(41.9, 58.1) |
| Rwanda | 15.9(12.6, 19.1) | 6.0(3.9, 8.1) | 9.9(6.1, 13.8) |
| Senegal | 11.8(9.2, 14.4) | 1.8(0.9, 2.7) | 10.0(7.3, 12.7) |
| Serbia | 95.6(93.2, 98.1) | 78.1(71.7, 84.6) | 17.5(10.5, 24.4) |
| Sierra Leone | 29.1(25.7, 32.4) | 11.2(9.1, 13.3) | 17.9(13.9, 21.8) |
| St. Lucia | 93.6(84.0, 103.2) | 67.5(48.5, 86.5) | 26.1(4.3, 48.0) |
| Suriname | 56.9(50.3, 63.5) | 17.6(13.1, 22.2) | 39.3(31.3, 47.3) |
| Swaziland | 30.0(22.6, 37.4) | 6.0(2.4, 9.6) | 24.0(15.8, 32.3) |
| São Tomé and Principe | 22.6(15.5, 29.7) | 6.0(2.4, 9.7) | 16.6(8.5, 24.6) |
| Thailand | 80.8(75.7, 86.0) | 46.6(40.3, 52.9) | 34.2(26.1, 42.3) |
| Togo | 13.6(10.5, 16.8) | 12.5(9.6, 15.4) | 1.1(-3.1, 5.4) |
| Tunisia | 76.3(70.2, 82.4) | 17.8(11.8, 23.7) | 58.5(50.0, 67.1) |
| Turkmenistan | 88.4(84.2, 92.6) | 80.4(75.9, 84.9) | 8.0(1.9, 14.2) |
| Uganda | 32.3(29.1, 35.5) | 9.5(7.7, 11.4) | 22.8(19.1, 26.5) |
| Ukraine | 88.2(84.1, 92.2) | 84.3(79.5, 89.2) | 3.8(-2.5, 10.2) |
| Uruguay | 89.4(83.5, 95.3) | 66.3(51.0, 81.6) | 23.1(6.7, 39.6) |
| Vietnam | 72.7(66.5, 78.9) | 21.7(15.5, 27.9) | 51.0(42.2, 59.8) |
| Zimbabwe | 28.0(24.5, 31.5) | 12.3(10.0, 14.6) | 15.7(11.5, 19.9) |
