# Supplementary material for: Maternal, paternal, and other caregivers’ stimulation in low- and- middle-income countries
Source: PLoS One. 2020 Jul 10;15(7):e0236107. doi: 10.1371/journal.pone.0236107 (PMC7351158; doi:10.1371/journal.pone.0236107)
Supplement: S7 Table — (DOCX) [file pone.0236107.s007.docx]

**S7 Table**. Wealth disparities in the percentage of children exposed to high paternal stimulation

| Country | Richest | Poorest | Difference (richest - poorest) |
| --- | --- | --- | --- |
| Afghanistan | 2.3(1.4, 3.3) | 3.2(1.9, 4.5) | 0.9(-0.8, 2.5) |
| Algeria | 10.3(7.8, 12.9) | 21.3(18.2, 24.4) | 10.9(6.9, 15.0) |
| Bangladesh | 4.3(3.3, 5.3) | 17.9(15.5, 20.4) | 13.6(10.9, 16.3) |
| Belarus | 26.3(18.4, 34.2) | 35.3(28.8, 41.8) | 9.0(-1.3, 19.3) |
| Belize | 25.7(19.5, 31.8) | 31.6(24.0, 39.1) | 5.9(-3.9, 15.7) |
| Benin | 3.8(2.4, 5.2) | 13.5(10.6, 16.3) | 9.7(6.5, 12.9) |
| Bosnia and Herzegovina | 19.2(14.0, 24.5) | 53.5(45.6, 61.4) | 34.3(24.7, 43.8) |
| Burundi | 2.5(1.7, 3.3) | 5.2(4.1, 6.4) | 2.7(1.3, 4.1) |
| Cameroon | 4.1(2.3, 5.8) | 6.3(4.0, 8.6) | 2.3(-0.6, 5.2) |
| Central African Republic | 5.8(3.9, 7.8) | 9.1(6.7, 11.5) | 3.3(0.2, 6.4) |
| Congo, Dem. Rep. | 0.4(0.1, 0.7) | 2.2(1.0, 3.5) | 1.8(0.5, 3.2) |
| Congo, Rep. | 6.9(5.1, 8.8) | 6.7(4.8, 8.7) | -0.2(-2.9, 2.5) |
| Costa Rica | 11.0(4.4, 17.6) | 13.9(7.2, 20.5) | 2.9(-6.5, 12.3) |
| Dominican Republic | 3.3(1.8, 4.8) | 10.7(8.2, 13.2) | 7.4(4.5, 10.3) |
| East Timor | 3.1(1.5, 4.7) | 6.2(4.0, 8.3) | 3.1(0.4, 5.7) |
| El Salvador | 6.1(3.4, 8.9) | 12.6(9.2, 16.1) | 6.5(2.1, 10.9) |
| Gambia | 0.4(-0.1, 0.8) | 2.5(1.0, 4.0) | 2.1(0.5, 3.7) |
| Ghana | 1.2(-0.3, 2.8) | 7.5(5.0, 10.1) | 6.3(3.3, 9.3) |
| Guinea | 2.0(0.8, 3.1) | 7.8(5.6, 10.0) | 5.9(3.4, 8.3) |
| Guinea-Bissau | 0.4(0.0, 0.7) | 0.4(-0.1, 1.0) | 0.1(-0.6, 0.7) |
| Guyana | 12.7(8.2, 17.2) | 23.8(18.4, 29.1) | 11.1(4.1, 18.1) |
| Iraq | 4.0(2.8, 5.2) | 13.8(11.3, 16.3) | 9.8(7.0, 12.5) |
| Ivory Coast | 3.5(2.1, 4.9) | 8.5(6.2, 10.8) | 5.0(2.4, 7.7) |
| Jamaica | 6.7(1.8, 11.6) | 25.7(15.5, 36.0) | 19.0(7.6, 30.4) |
| Jordan | 13.8(10.2, 17.5) | 27.0(21.8, 32.3) | 13.2(6.8, 19.6) |
| Kazakhstan | 6.2(1.0, 11.4) | 11.3(7.9, 14.7) | 5.2(-1.1, 11.4) |
| Kosovo | 9.3(3.5, 15.1) | 5.2(1.1, 9.3) | -4.1(-11.2, 3.0) |
| Lao PDR | 5.5(3.9, 7.0) | 19.3(16.5, 22.2) | 13.9(10.6, 17.1) |
| Kyrgyzstan | 2.7(0.8, 4.7) | 4.9(2.1, 7.8) | 2.2(-1.3, 5.7) |
| Lebanon | 1.4(-0.4, 3.1) | 15.0(9.0, 21.0) | 13.7(7.4, 20.0) |
| Macedonia | 9.3(-1.4, 20.0) | 28.8(19.2, 38.4) | 19.5(5.0, 34.0) |
| Malawi | 1.3(0.6, 2.0) | 4.5(3.1, 5.9) | 3.2(1.7, 4.8) |
| Maldives | 9.7(5.9, 13.5) | 32.0(24.4, 39.6) | 22.3(13.8, 30.8) |
| Mali | 5.7(4.1, 7.4) | 6.7(5.1, 8.4) | 1.0(-1.3, 3.3) |
| Mauritania | 2.8(1.7, 3.9) | 10.1(7.4, 12.7) | 7.3(4.4, 10.2) |
| Mexico | 5.9(2.9, 8.9) | 18.5(10.1, 26.9) | 12.6(3.7, 21.5) |
| Moldova | 4.4(0.7, 8.0) | 24.9(17.4, 32.5) | 20.6(12.2, 29.0) |
| Mongolia | 7.2(4.8, 9.7) | 17.1(13.5, 20.7) | 9.8(5.5, 14.2) |
| Montenegro | 25.6(17.0, 34.3) | 55.8(44.9, 66.7) | 30.2(16.2, 44.1) |
| Nepal | 8.3(5.2, 11.4) | 14.7(10.9, 18.4) | 6.4(1.5, 11.3) |
| Nigeria | 5.2(4.2, 6.2) | 23.1(20.9, 25.2) | 17.9(15.5, 20.2) |
| Palestine | 8.4(6.3, 10.5) | 16.8(13.9, 19.8) | 8.4(4.8, 12.1) |
| Panama | 4.4(2.0, 6.8) | 21.4(15.2, 27.6) | 17.0(10.4, 23.7) |
| Paraguay | 9.9(4.9, 14.8) | 30.5(24.1, 36.8) | 20.6(12.5, 28.7) |
| Rwanda | 1.2(0.2, 2.1) | 3.3(1.8, 4.7) | 2.1(0.4, 3.9) |
| Senegal | 0.7(-0.1, 1.5) | 1.5(0.6, 2.4) | 0.8(-0.4, 2.0) |
| Serbia | 21.0(14.7, 27.4) | 41.8(29.0, 54.6) | 20.8(6.5, 35.1) |
| Sierra Leone | 6.7(4.9, 8.4) | 11.0(8.7, 13.4) | 4.4(1.4, 7.3) |
| St. Lucia | 1.9(-1.9, 5.7) | 34.0(14.6, 53.5) | 32.1(11.9, 52.4) |
| Suriname | 0.4(-0.3, 1.1) | 14.5(9.9, 19.1) | 14.2(9.5, 18.8) |
| Swaziland | .(., .) | 5.3(2.2, 8.4) | 5.3(2.2, 8.4) |
| São Tomé and Principe | 0.6(-0.5, 1.7) | 5.5(1.5, 9.6) | 5.0(0.7, 9.2) |
| Thailand | 23.6(18.2, 29.1) | 49.6(42.5, 56.7) | 26.0(17.0, 34.9) |
| Togo | 4.8(2.9, 6.7) | 3.8(2.0, 5.7) | -1.0(-3.6, 1.6) |
| Tunisia | 8.3(4.2, 12.4) | 37.7(30.5, 44.9) | 29.4(21.1, 37.7) |
| Turkmenistan | 9.2(5.9, 12.6) | 17.2(12.9, 21.5) | 8.0(2.5, 13.5) |
| Uganda | 0.8(0.2, 1.3) | 5.1(3.5, 6.8) | 4.3(2.6, 6.1) |
| Ukraine | 17.9(12.2, 23.6) | 38.2(31.0, 45.4) | 20.3(11.1, 29.5) |
| Uruguay | 28.8(13.4, 44.3) | 47.0(35.7, 58.3) | 18.2(-1.0, 37.4) |
| Vietnam | 2.7(0.1, 5.2) | 29.6(23.3, 36.0) | 27.0(20.1, 33.9) |
| Zimbabwe | 1.0(0.3, 1.6) | 6.1(4.3, 8.0) | 5.2(3.2, 7.2) |
