# Supplementary material for: Maternal, paternal, and other caregivers’ stimulation in low- and- middle-income countries
Source: PLoS One. 2020 Jul 10;15(7):e0236107. doi: 10.1371/journal.pone.0236107 (PMC7351158; doi:10.1371/journal.pone.0236107)
Supplement: S8 Table — (DOCX) [file pone.0236107.s008.docx]

**S8 Table**. Wealth disparities in the percentage of children exposed to high stimulation by other caregivers

| Country | Richest | Poorest | Difference (richest - poorest) |
| --- | --- | --- | --- |
| Afghanistan | 8.2(6.8, 9.6) | 23.6(21.0, 26.2) | 15.4(12.4, 18.4) |
| Algeria | 16.3(13.4, 19.1) | 25.6(22.4, 28.7) | 9.3(5.0, 13.5) |
| Bangladesh | 21.8(19.6, 24.0) | 27.6(24.9, 30.3) | 5.8(2.3, 9.2) |
| Belarus | 14.4(8.4, 20.5) | 12.2(7.7, 16.7) | -2.2(-9.7, 5.3) |
| Belize | 29.7(23.2, 36.3) | 26.3(18.6, 34.0) | -3.4(-13.6, 6.7) |
| Benin | 2.4(1.3, 3.5) | 13.9(10.6, 17.2) | 11.5(8.0, 15.0) |
| Bosnia and Herzegovina | 35.4(27.7, 43.1) | 29.6(22.9, 36.3) | -5.8(-16.0, 4.5) |
| Burundi | 30.5(28.4, 32.6) | 36.6(34.0, 39.2) | 6.1(2.7, 9.5) |
| Cameroon | 29.7(25.5, 33.8) | 26.5(22.4, 30.7) | -3.1(-9.0, 2.7) |
| Central African Republic | 36.0(31.7, 40.3) | 27.3(23.7, 30.9) | -8.7(-14.3, -3.2) |
| Congo, Dem. Rep. | 11.1(8.3, 13.8) | 13.4(11.1, 15.6) | 2.3(-1.3, 5.9) |
| Congo, Rep. | 13.0(10.2, 15.8) | 25.3(21.7, 28.9) | 12.3(7.7, 16.8) |
| Costa Rica | 12.2(4.2, 20.1) | 18.6(9.8, 27.4) | 6.4(-5.5, 18.3) |
| Dominican Republic | 10.6(8.6, 12.6) | 19.6(16.5, 22.7) | 9.0(5.3, 12.7) |
| East Timor | 4.6(2.7, 6.5) | 7.1(4.4, 9.8) | 2.5(-0.8, 5.8) |
| El Salvador | 5.4(3.6, 7.2) | 8.7(5.4, 12.0) | 3.3(-0.5, 7.0) |
| Gambia | 39.2(34.7, 43.6) | 33.5(29.4, 37.6) | -5.7(-11.7, 0.4) |
| Ghana | 11.6(8.1, 15.0) | 24.7(20.3, 29.1) | 13.1(7.5, 18.7) |
| Guinea | 8.9(6.6, 11.2) | 24.3(20.6, 27.9) | 15.3(11.0, 19.7) |
| Guinea-Bissau | 20.9(17.1, 24.8) | 21.1(17.3, 24.8) | 0.1(-5.2, 5.5) |
| Guyana | 35.8(29.5, 42.2) | 31.7(25.9, 37.5) | -4.1(-12.8, 4.5) |
| Iraq | 11.5(8.9, 14.0) | 17.1(12.7, 21.6) | 5.6(0.5, 10.8) |
| Ivory Coast | 3.7(2.3, 5.2) | 15.9(12.8, 19.1) | 12.2(8.7, 15.7) |
| Jamaica | 26.0(17.5, 34.4) | 30.3(19.2, 41.4) | 4.4(-9.6, 18.4) |
| Jordan | 9.7(6.7, 12.7) | 6.7(3.9, 9.6) | -3.0(-7.2, 1.1) |
| Kazakhstan | 30.3(24.6, 36.0) | 21.2(16.8, 25.6) | -9.1(-16.4, -1.9) |
| Kosovo | 9.9(5.1, 14.7) | 11.5(5.9, 17.0) | 1.6(-5.8, 9.0) |
| Lao PDR | 10.7(8.5, 13.0) | 22.2(19.2, 25.1) | 11.5(7.8, 15.2) |
| Kyrgyzstan | 19.4(14.6, 24.2) | 19.2(14.0, 24.4) | -0.2(-7.3, 6.9) |
| Lebanon | 17.3(10.8, 23.8) | 18.9(12.4, 25.5) | 1.7(-7.6, 10.9) |
| Macedonia | 14.0(7.0, 21.1) | 27.6(17.7, 37.5) | 13.6(1.3, 25.8) |
| Malawi | 8.9(7.0, 10.8) | 13.3(11.1, 15.6) | 4.5(1.5, 7.4) |
| Maldives | 18.6(13.3, 23.8) | 25.1(18.0, 32.2) | 6.6(-2.3, 15.4) |
| Mali | 17.1(14.8, 19.4) | 28.5(25.7, 31.4) | 11.4(7.8, 15.1) |
| Mauritania | 13.5(11.1, 15.8) | 25.0(21.2, 28.7) | 11.5(7.0, 15.9) |
| Mexico | 8.3(5.1, 11.5) | 15.7(7.3, 24.1) | 7.3(-1.6, 16.3) |
| Moldova | 23.5(16.3, 30.8) | 16.4(10.2, 22.5) | -7.2(-16.7, 2.4) |
| Mongolia | 8.7(6.1, 11.3) | 19.0(15.3, 22.8) | 10.4(5.8, 15.0) |
| Montenegro | 36.0(26.7, 45.3) | 27.4(17.9, 37.0) | -8.6(-22.0, 4.8) |
| Nepal | 21.0(16.4, 25.6) | 30.1(25.3, 34.9) | 9.1(2.4, 15.7) |
| Nigeria | 26.5(24.4, 28.6) | 35.1(32.7, 37.5) | 8.6(5.4, 11.8) |
| Palestine | 12.5(9.9, 15.1) | 19.4(16.3, 22.4) | 6.8(2.8, 10.8) |
| Panama | 13.9(9.5, 18.3) | 17.6(12.2, 23.0) | 3.7(-3.2, 10.7) |
| Paraguay | 9.4(6.1, 12.7) | 22.5(17.0, 28.0) | 13.1(6.7, 19.6) |
| Rwanda | 14.2(11.3, 17.1) | 25.1(21.2, 29.1) | 11.0(6.0, 15.9) |
| Senegal | 6.9(5.2, 8.5) | 17.0(14.0, 20.0) | 10.1(6.7, 13.5) |
| Serbia | 16.4(9.9, 22.9) | 21.8(12.1, 31.6) | 5.4(-6.3, 17.2) |
| Sierra Leone | 2.0(1.0, 3.1) | 5.8(4.1, 7.6) | 3.8(1.8, 5.8) |
| St. Lucia | 52.5(31.7, 73.3) | 43.0(22.9, 63.0) | -9.5(-39.1, 20.1) |
| Suriname | 15.9(11.1, 20.7) | 26.6(20.8, 32.5) | 10.7(3.2, 18.3) |
| Swaziland | 14.1(9.0, 19.1) | 14.9(9.7, 20.0) | 0.8(-6.5, 8.0) |
| São Tomé and Principe | 26.8(19.4, 34.3) | 33.8(25.9, 41.7) | 6.9(-3.9, 17.8) |
| Thailand | 52.5(46.3, 58.8) | 55.7(48.8, 62.5) | 3.1(-6.2, 12.4) |
| Togo | 17.7(14.2, 21.3) | 8.7(6.1, 11.4) | -9.0(-13.4, -4.5) |
| Tunisia | 13.8(8.6, 18.9) | 11.9(7.3, 16.5) | -1.9(-8.8, 5.0) |
| Turkmenistan | 8.3(5.1, 11.6) | 12.5(8.3, 16.8) | 4.2(-1.2, 9.5) |
| Uganda | 17.6(15.2, 20.0) | 24.3(21.5, 27.1) | 6.7(3.0, 10.4) |
| Ukraine | 20.2(15.2, 25.1) | 26.5(20.5, 32.5) | 6.4(-1.4, 14.1) |
| Uruguay | 38.7(21.9, 55.5) | 32.7(22.6, 42.8) | -6.0(-25.7, 13.7) |
| Vietnam | 16.3(10.8, 21.9) | 27.5(21.3, 33.6) | 11.1(2.8, 19.4) |
| Zimbabwe | 17.4(14.8, 20.0) | 18.1(15.3, 21.0) | 0.7(-3.1, 4.6) |
