# Supplementary material for: Maternal, paternal, and other caregivers’ stimulation in low- and- middle-income countries
Source: PLoS One. 2020 Jul 10;15(7):e0236107. doi: 10.1371/journal.pone.0236107 (PMC7351158; doi:10.1371/journal.pone.0236107)
Supplement: S9 Table — (DOCX) [file pone.0236107.s009.docx]

**S9 Table**. Sex disparities in the percentage of children exposed to high maternal stimulation

| Country | Male | Female | Difference (Male - Female) |
| --- | --- | --- | --- |
| Afghanistan | 4.4(3.6, 5.3) | 4.6(3.8, 5.4) | -0.1(-1.3, 1.0) |
| Algeria | 38.7(36.3, 41.1) | 38.9(36.4, 41.3) | -0.2(-3.6, 3.2) |
| Argentina | 67.1(63.5, 70.7) | 73.6(70.3, 76.8) | -6.5(-11.3, -1.6) |
| Bangladesh | 40.8(38.9, 42.6) | 40.8(38.9, 42.8) | -0.0(-2.7, 2.7) |
| Belarus | 82.8(78.9, 86.7) | 87.8(84.7, 90.9) | -5.0(-10.0, -0.0) |
| Belize | 68.4(63.8, 73.0) | 69.4(64.3, 74.5) | -1.0(-7.8, 5.8) |
| Benin | 13.2(11.6, 14.9) | 13.8(12.2, 15.4) | -0.5(-2.8, 1.7) |
| Bosnia and Herzegovina | 86.6(82.8, 90.3) | 86.0(82.2, 89.7) | 0.6(-4.7, 5.9) |
| Burundi | 14.6(13.5, 15.7) | 16.4(15.3, 17.5) | -1.8(-3.4, -0.2) |
| Cameroon | 13.3(11.3, 15.4) | 14.0(11.9, 16.0) | -0.6(-3.5, 2.2) |
| Central African Republic | 20.5(18.3, 22.7) | 23.2(21.0, 25.5) | -2.7(-5.9, 0.4) |
| Congo, Dem. Rep. | 4.7(3.7, 5.8) | 4.7(3.7, 5.6) | 0.0(-1.4, 1.5) |
| Congo, Rep. | 24.7(21.8, 27.6) | 29.0(25.7, 32.2) | -4.3(-8.6, 0.1) |
| Costa Rica | 50.4(42.0, 58.7) | 44.4(36.3, 52.6) | 5.9(-5.8, 17.6) |
| Dominican Republic | 31.8(29.6, 34.1) | 31.8(29.6, 34.0) | -0.0(-3.1, 3.1) |
| East Timor | 19.6(17.1, 22.1) | 19.9(17.4, 22.5) | -0.4(-3.9, 3.2) |
| El Salvador | 37.8(34.6, 41.0) | 43.4(40.0, 46.7) | -5.6(-10.2, -0.9) |
| Gambia | 7.3(5.9, 8.8) | 6.1(4.9, 7.4) | 1.2(-0.7, 3.1) |
| Ghana | 11.3(8.8, 13.9) | 11.3(8.9, 13.7) | 0.0(-3.4, 3.5) |
| Guinea | 14.8(12.9, 16.7) | 14.6(12.6, 16.5) | 0.2(-2.5, 3.0) |
| Guinea-Bissau | 3.0(1.9, 4.2) | 2.9(1.7, 4.1) | 0.1(-1.6, 1.8) |
| Guyana | 56.4(52.5, 60.4) | 56.7(52.6, 60.7) | -0.3(-5.9, 5.4) |
| Ivory Coast | 12.1(10.1, 14.1) | 13.9(11.7, 16.0) | -1.8(-4.7, 1.2) |
| Iraq | 22.7(20.1, 25.3) | 24.5(21.6, 27.3) | -1.8(-5.6, 2.1) |
| Jamaica | 60.7(53.9, 67.5) | 61.4(55.0, 67.9) | -0.7(-10.1, 8.6) |
| Jordan | 64.8(61.4, 68.2) | 68.7(65.3, 72.2) | -3.9(-8.8, 1.0) |
| Kazakhstan | 51.1(46.8, 55.4) | 51.5(46.9, 56.1) | -0.4(-6.7, 5.9) |
| Kosovo | 42.8(37.4, 48.3) | 44.6(38.9, 50.3) | -1.8(-9.7, 6.1) |
| Lao PDR | 19.5(17.6, 21.3) | 20.5(18.6, 22.4) | -1.0(-3.6, 1.6) |
| Kyrgyzstan | 28.9(25.4, 32.5) | 31.8(28.1, 35.5) | -2.9(-8.0, 2.3) |
| Lebanon | 54.0(48.9, 59.1) | 52.1(46.6, 57.6) | 1.9(-5.6, 9.4) |
| Macedonia | 64.8(58.2, 71.4) | 55.9(48.8, 63.1) | 8.9(-0.9, 18.6) |
| Malawi | 8.9(7.7, 10.1) | 10.8(9.5, 12.0) | -1.9(-3.6, -0.1) |
| Maldives | 86.7(83.2, 90.2) | 87.1(83.4, 90.8) | -0.4(-5.5, 4.7) |
| Mali | 19.1(17.6, 20.6) | 19.3(17.8, 20.9) | -0.2(-2.4, 1.9) |
| Mauritania | 21.3(19.2, 23.5) | 20.1(18.1, 22.1) | 1.3(-1.7, 4.2) |
| Mexico | 58.8(55.0, 62.5) | 66.0(58.9, 73.0) | -7.2(-15.1, 0.8) |
| Moldova | 67.4(62.2, 72.7) | 70.9(65.3, 76.4) | -3.4(-11.1, 4.2) |
| Mongolia | 28.0(25.2, 30.7) | 29.6(26.8, 32.3) | -1.6(-5.5, 2.2) |
| Montenegro | 92.0(88.9, 95.1) | 92.0(88.8, 95.3) | -0.1(-4.5, 4.4) |
| Nepal | 31.4(28.0, 34.8) | 29.8(26.4, 33.3) | 1.5(-3.3, 6.4) |
| Nigeria | 27.7(26.3, 29.1) | 28.8(27.4, 30.3) | -1.2(-3.2, 0.9) |
| Palestine | 55.2(52.8, 57.7) | 55.6(53.1, 58.2) | -0.4(-3.9, 3.1) |
| Panama | 54.5(49.5, 59.5) | 53.5(48.1, 58.8) | 1.0(-6.3, 8.4) |
| Paraguay | 41.3(37.2, 45.4) | 46.3(42.0, 50.7) | -5.0(-11.0, 0.9) |
| Rwanda | 10.6(9.0, 12.3) | 11.9(10.1, 13.7) | -1.3(-3.7, 1.2) |
| Senegal | 5.6(4.4, 6.8) | 7.0(5.5, 8.5) | -1.4(-3.3, 0.6) |
| Serbia | 90.0(86.9, 93.0) | 89.2(86.2, 92.2) | 0.8(-3.5, 5.1) |
| Sierra Leone | 16.2(14.5, 18.0) | 19.1(17.2, 20.9) | -2.8(-5.4, -0.3) |
| St. Lucia | 69.1(56.2, 82.1) | 72.6(60.5, 84.6) | -3.5(-21.3, 14.4) |
| Suriname | 37.1(32.5, 41.7) | 42.8(38.2, 47.4) | -5.7(-12.2, 0.8) |
| Swaziland | 12.1(8.6, 15.5) | 21.3(17.1, 25.6) | -9.3(-14.8, -3.8) |
| São Tomé and Principe | 17.2(13.0, 21.4) | 14.7(10.7, 18.8) | 2.4(-3.4, 8.3) |
| Thailand | 63.7(60.1, 67.3) | 63.5(59.3, 67.7) | 0.2(-5.4, 5.7) |
| Togo | 6.8(5.4, 8.2) | 10.1(8.3, 11.8) | -3.3(-5.5, -1.0) |
| Tunisia | 45.4(40.7, 50.0) | 56.1(51.1, 61.1) | -10.7(-17.5, -3.9) |
| Turkmenistan | 80.9(78.0, 83.8) | 83.3(80.3, 86.2) | -2.4(-6.6, 1.8) |
| Uganda | 17.2(15.5, 18.8) | 19.8(18.1, 21.5) | -2.7(-5.0, -0.3) |
| Ukraine | 84.2(80.9, 87.5) | 86.2(82.3, 90.0) | -2.0(-7.1, 3.1) |
| Uruguay | 77.6(69.2, 86.1) | 77.8(70.1, 85.5) | -0.2(-11.6, 11.2) |
| Vietnam | 45.6(41.1, 50.1) | 45.9(41.4, 50.4) | -0.3(-6.7, 6.1) |
| Zimbabwe | 17.1(15.4, 18.9) | 18.3(16.5, 20.1) | -1.2(-3.7, 1.3) |
