# Supplementary material for: Maternal, paternal, and other caregivers’ stimulation in low- and- middle-income countries
Source: PLoS One. 2020 Jul 10;15(7):e0236107. doi: 10.1371/journal.pone.0236107 (PMC7351158; doi:10.1371/journal.pone.0236107)
Supplement: S10 Table — (DOCX) [file pone.0236107.s010.docx]

**S10 Table**. Sex disparities in the percentage of children exposed to high paternal stimulation

| Country | Male | Female | Difference (Male - Female) |
| --- | --- | --- | --- |
| Afghanistan | 4.6(3.7, 5.5) | 3.3(2.5, 4.0) | 1.3(0.2, 2.5) |
| Algeria | 19.1(17.1, 21.2) | 15.5(13.6, 17.4) | 3.6(0.8, 6.4) |
| Argentina | 20.6(17.7, 23.5) | 24.2(20.8, 27.6) | -3.6(-8.1, 0.8) |
| Bangladesh | 10.5(9.3, 11.7) | 9.7(8.5, 10.9) | 0.8(-1.0, 2.5) |
| Belarus | 24.5(20.3, 28.7) | 32.8(28.1, 37.5) | -8.3(-14.6, -2.0) |
| Belize | 25.4(21.0, 29.7) | 22.5(18.3, 26.6) | 2.9(-3.1, 8.9) |
| Benin | 6.6(5.4, 7.8) | 4.0(3.1, 4.8) | 2.6(1.1, 4.1) |
| Bosnia and Herzegovina | 29.7(24.6, 34.9) | 32.0(27.6, 36.4) | -2.3(-9.0, 4.5) |
| Burundi | 3.1(2.5, 3.6) | 2.1(1.7, 2.6) | 0.9(0.2, 1.7) |
| Cameroon | 3.7(2.5, 5.0) | 3.8(2.7, 4.9) | -0.1(-1.7, 1.6) |
| Central African Republic | 10.3(8.6, 12.0) | 7.0(5.6, 8.4) | 3.3(1.1, 5.5) |
| Congo, Dem. Rep. | 1.7(0.9, 2.5) | 1.1(0.5, 1.6) | 0.6(-0.3, 1.6) |
| Congo, Rep. | 6.9(5.5, 8.4) | 5.6(4.1, 7.1) | 1.4(-0.7, 3.4) |
| Costa Rica | 10.2(6.1, 14.3) | 13.3(8.3, 18.4) | -3.1(-9.6, 3.4) |
| Dominican Republic | 6.6(5.3, 7.9) | 6.6(5.5, 7.6) | 0.0(-1.7, 1.7) |
| East Timor | 5.3(4.0, 6.5) | 4.4(3.2, 5.6) | 0.9(-0.9, 2.6) |
| El Salvador | 7.6(6.0, 9.2) | 8.4(6.4, 10.4) | -0.8(-3.3, 1.8) |
| Gambia | 1.3(0.6, 2.1) | 0.5(0.1, 0.9) | 0.8(-0.0, 1.7) |
| Ghana | 3.8(2.3, 5.3) | 3.0(1.8, 4.2) | 0.8(-1.1, 2.7) |
| Guinea | 5.0(3.8, 6.2) | 3.5(2.5, 4.4) | 1.5(0.0, 3.0) |
| Guinea-Bissau | 0.3(-0.0, 0.7) | 0.3(0.0, 0.6) | 0.0(-0.5, 0.5) |
| Guyana | 16.2(13.2, 19.2) | 16.0(13.1, 19.0) | 0.2(-4.0, 4.4) |
| Iraq | 11.2(9.0, 13.4) | 9.2(7.5, 11.0) | 1.9(-0.9, 4.8) |
| Ivory Coast | 5.5(4.2, 6.8) | 4.4(3.1, 5.6) | 1.2(-0.6, 2.9) |
| Jamaica | 12.2(7.9, 16.5) | 15.2(10.3, 20.0) | -3.0(-9.5, 3.5) |
| Jordan | 20.8(17.9, 23.8) | 20.5(17.3, 23.7) | 0.3(-4.1, 4.7) |
| Kazakhstan | 7.3(5.4, 9.2) | 6.1(3.4, 8.9) | 1.1(-2.2, 4.5) |
| Kosovo | 7.6(4.4, 10.7) | 4.6(2.2, 6.9) | 3.0(-1.0, 7.0) |
| Lao PDR | 11.9(10.4, 13.4) | 10.0(8.6, 11.5) | 1.9(-0.2, 4.0) |
| Kyrgyzstan | 3.2(1.9, 4.6) | 2.5(1.3, 3.7) | 0.7(-1.1, 2.6) |
| Lebanon | 10.1(7.0, 13.2) | 8.3(5.2, 11.3) | 1.9(-2.5, 6.2) |
| Macedonia | 17.1(11.0, 23.3) | 21.0(15.6, 26.3) | -3.8(-12.0, 4.3) |
| Malawi | 3.2(2.4, 3.9) | 3.0(2.2, 3.9) | 0.1(-1.0, 1.3) |
| Maldives | 24.3(19.3, 29.3) | 22.1(17.1, 27.0) | 2.2(-4.8, 9.3) |
| Mali | 6.2(5.2, 7.1) | 4.1(3.2, 4.9) | 2.1(0.8, 3.4) |
| Mauritania | 5.2(4.0, 6.4) | 4.3(3.3, 5.3) | 0.9(-0.7, 2.4) |
| Mexico | 14.5(11.5, 17.6) | 14.7(10.1, 19.2) | -0.1(-5.6, 5.4) |
| Moldova | 14.3(10.5, 18.1) | 12.0(8.4, 15.6) | 2.3(-3.0, 7.5) |
| Mongolia | 9.5(7.7, 11.3) | 10.3(8.5, 12.1) | -0.7(-3.3, 1.8) |
| Montenegro | 45.8(39.3, 52.2) | 45.2(38.3, 52.2) | 0.5(-9.0, 10.1) |
| Nepal | 9.4(7.4, 11.4) | 10.9(8.6, 13.1) | -1.5(-4.5, 1.5) |
| Nigeria | 11.7(10.7, 12.7) | 10.1(9.2, 11.0) | 1.6(0.2, 2.9) |
| Palestine | 12.2(10.6, 13.8) | 12.4(10.7, 14.0) | -0.2(-2.5, 2.1) |
| Panama | 16.4(12.2, 20.7) | 11.2(7.4, 15.1) | 5.2(-0.5, 10.9) |
| Paraguay | 15.9(12.8, 19.0) | 17.7(14.0, 21.3) | -1.8(-6.6, 3.0) |
| Rwanda | 2.1(1.4, 2.9) | 2.1(1.3, 2.8) | 0.1(-1.0, 1.2) |
| Senegal | 0.9(0.4, 1.5) | 0.7(0.2, 1.1) | 0.3(-0.4, 1.0) |
| Serbia | 40.8(32.8, 48.8) | 31.9(25.8, 38.1) | 8.9(-1.2, 19.0) |
| Sierra Leone | 8.5(7.1, 9.9) | 6.2(5.1, 7.3) | 2.3(0.6, 4.1) |
| St. Lucia | 22.9(11.2, 34.6) | 21.6(10.6, 32.7) | 1.3(-15.0, 17.6) |
| Suriname | 7.7(4.9, 10.4) | 7.9(5.3, 10.5) | -0.2(-4.0, 3.6) |
| Swaziland | 1.8(0.5, 3.2) | 1.8(0.5, 3.1) | 0.0(-1.9, 1.9) |
| São Tomé and Principe | 2.9(1.1, 4.7) | 3.2(1.5, 5.0) | -0.3(-2.9, 2.2) |
| Thailand | 33.2(29.5, 36.8) | 35.7(31.4, 40.0) | -2.6(-8.2, 3.1) |
| Togo | 3.4(2.3, 4.5) | 2.0(1.3, 2.8) | 1.4(0.1, 2.7) |
| Tunisia | 19.6(15.8, 23.4) | 20.0(15.8, 24.3) | -0.4(-6.1, 5.2) |
| Turkmenistan | 16.2(13.4, 19.0) | 14.0(11.3, 16.6) | 2.2(-1.6, 6.1) |
| Uganda | 2.7(2.0, 3.4) | 2.8(2.1, 3.5) | -0.1(-1.1, 0.9) |
| Ukraine | 25.5(21.7, 29.4) | 28.1(23.8, 32.4) | -2.6(-8.3, 3.2) |
| Uruguay | 31.3(20.5, 42.2) | 28.4(19.4, 37.3) | 2.9(-11.1, 17.0) |
| Vietnam | 14.5(11.3, 17.6) | 15.9(12.5, 19.3) | -1.4(-6.0, 3.2) |
| Zimbabwe | 3.1(2.3, 3.9) | 2.2(1.5, 2.8) | 0.9(-0.1, 2.0) |
