# Supplementary material for: Maternal, paternal, and other caregivers’ stimulation in low- and- middle-income countries
Source: PLoS One. 2020 Jul 10;15(7):e0236107. doi: 10.1371/journal.pone.0236107 (PMC7351158; doi:10.1371/journal.pone.0236107)
Supplement: S11 Table — (DOCX) [file pone.0236107.s011.docx]

**S11 Table**. Sex disparities in the percentage of children exposed to high stimulation by other caregivers

| Country | Male | Female | Difference (Male - Female) |
| --- | --- | --- | --- |
| Afghanistan | 14.6(13.2, 16.0) | 14.9(13.5, 16.2) | -0.3(-2.2, 1.6) |
| Algeria | 21.9(19.8, 23.9) | 23.5(21.4, 25.6) | -1.6(-4.6, 1.3) |
| Argentina | 16.9(14.2, 19.7) | 18.4(15.5, 21.3) | -1.4(-5.4, 2.5) |
| Bangladesh | 25.1(23.5, 26.7) | 25.1(23.4, 26.8) | -0.0(-2.3, 2.3) |
| Belarus | 13.4(9.9, 16.9) | 16.6(12.7, 20.5) | -3.2(-8.5, 2.0) |
| Belize | 24.1(20.1, 28.2) | 26.8(22.1, 31.6) | -2.7(-8.9, 3.6) |
| Benin | 6.1(4.9, 7.3) | 6.9(5.6, 8.1) | -0.8(-2.6, 1.0) |
| Bosnia and Herzegovina | 30.0(24.3, 35.6) | 34.7(29.8, 39.6) | -4.7(-12.2, 2.8) |
| Burundi | 30.9(29.5, 32.3) | 30.2(28.8, 31.6) | 0.7(-1.2, 2.7) |
| Cameroon | 24.9(22.2, 27.5) | 23.6(21.1, 26.2) | 1.2(-2.5, 4.9) |
| Central African Republic | 34.4(31.7, 37.1) | 35.1(32.5, 37.7) | -0.8(-4.5, 3.0) |
| Congo, Dem. Rep. | 11.8(10.3, 13.3) | 11.8(10.3, 13.3) | -0.0(-2.2, 2.1) |
| Congo, Rep. | 20.5(17.8, 23.2) | 19.4(16.7, 22.2) | 1.1(-2.8, 5.0) |
| Costa Rica | 17.9(10.6, 25.1) | 13.9(9.0, 18.7) | 4.0(-4.7, 12.7) |
| Dominican Republic | 15.7(13.9, 17.5) | 17.2(15.4, 19.0) | -1.5(-4.0, 1.0) |
| East Timor | 4.8(3.4, 6.1) | 4.3(2.9, 5.6) | 0.5(-1.4, 2.4) |
| El Salvador | 6.5(4.8, 8.2) | 7.0(5.4, 8.6) | -0.5(-2.9, 1.9) |
| Gambia | 36.1(33.2, 38.9) | 36.7(34.0, 39.5) | -0.7(-4.7, 3.3) |
| Ghana | 18.1(15.1, 21.0) | 19.3(16.2, 22.5) | -1.3(-5.6, 3.0) |
| Guinea | 13.7(11.9, 15.6) | 13.3(11.5, 15.2) | 0.4(-2.2, 3.0) |
| Guinea-Bissau | 23.1(20.5, 25.7) | 13.2(11.2, 15.2) | 9.9(6.7, 13.2) |
| Guyana | 33.2(29.5, 37.0) | 33.6(29.7, 37.5) | -0.4(-5.8, 5.0) |
| Iraq | 13.7(11.2, 16.2) | 17.0(14.2, 19.8) | -3.3(-7.1, 0.4) |
| Ivory Coast | 8.2(6.6, 9.9) | 9.2(7.5, 10.9) | -0.9(-3.3, 1.4) |
| Jamaica | 33.9(27.7, 40.1) | 43.3(36.8, 49.8) | -9.4(-18.4, -0.4) |
| Jordan | 8.1(6.1, 10.2) | 7.5(5.7, 9.3) | 0.6(-2.1, 3.4) |
| Kazakhstan | 26.8(23.0, 30.6) | 28.7(24.7, 32.8) | -1.9(-7.5, 3.6) |
| Kosovo | 12.4(8.9, 15.9) | 17.6(13.2, 21.9) | -5.2(-10.8, 0.5) |
| Lao PDR | 14.8(13.1, 16.4) | 15.7(13.9, 17.4) | -0.9(-3.2, 1.5) |
| Kyrgyzstan | 24.7(21.4, 28.1) | 23.9(20.4, 27.4) | 0.8(-4.0, 5.6) |
| Lebanon | 15.8(12.0, 19.6) | 20.9(16.3, 25.4) | -5.1(-11.0, 0.8) |
| Macedonia | 21.7(16.4, 26.9) | 19.6(14.3, 25.0) | 2.0(-5.5, 9.6) |
| Malawi | 10.3(9.1, 11.6) | 10.4(9.1, 11.8) | -0.1(-2.0, 1.7) |
| Maldives | 19.7(15.2, 24.1) | 22.3(17.5, 27.1) | -2.7(-9.2, 3.9) |
| Mali | 23.4(21.8, 25.1) | 24.6(22.8, 26.3) | -1.1(-3.5, 1.2) |
| Mauritania | 19.1(17.1, 21.1) | 17.7(15.8, 19.6) | 1.4(-1.4, 4.2) |
| Mexico | 9.8(7.7, 11.9) | 12.4(8.3, 16.6) | -2.6(-7.3, 2.0) |
| Moldova | 18.3(14.1, 22.5) | 28.4(22.9, 33.8) | -10.1(-17.0, -3.2) |
| Mongolia | 14.5(12.4, 16.7) | 14.1(12.0, 16.2) | 0.4(-2.6, 3.4) |
| Montenegro | 32.2(26.1, 38.2) | 31.0(24.7, 37.3) | 1.2(-7.5, 10.0) |
| Nepal | 31.1(27.6, 34.5) | 28.2(24.8, 31.6) | 2.9(-2.0, 7.7) |
| Nigeria | 30.6(29.2, 32.1) | 31.6(30.0, 33.1) | -1.0(-3.1, 1.2) |
| Palestine | 14.4(12.7, 16.1) | 15.5(13.7, 17.3) | -1.1(-3.6, 1.4) |
| Panama | 15.8(12.2, 19.5) | 16.0(11.9, 20.1) | -0.1(-5.6, 5.3) |
| Paraguay | 18.6(15.2, 22.0) | 16.7(13.3, 20.0) | 1.9(-2.9, 6.7) |
| Rwanda | 21.3(19.1, 23.5) | 19.0(16.9, 21.1) | 2.3(-0.8, 5.3) |
| Senegal | 12.3(10.6, 14.0) | 13.5(11.7, 15.3) | -1.3(-3.8, 1.2) |
| Serbia | 20.0(14.2, 25.8) | 16.2(12.1, 20.4) | 3.8(-3.4, 10.9) |
| Sierra Leone | 3.1(2.2, 4.0) | 3.3(2.4, 4.1) | -0.2(-1.4, 1.1) |
| St. Lucia | 39.3(25.5, 53.0) | 42.9(29.6, 56.3) | -3.6(-23.0, 15.7) |
| Suriname | 23.4(19.2, 27.5) | 23.6(19.8, 27.5) | -0.3(-5.9, 5.4) |
| Swaziland | 15.6(12.3, 19.0) | 18.2(14.7, 21.7) | -2.6(-7.5, 2.3) |
| São Tomé and Principe | 30.7(25.7, 35.7) | 29.0(24.1, 33.9) | 1.7(-5.3, 8.7) |
| Thailand | 52.3(48.4, 56.2) | 53.0(48.7, 57.4) | -0.7(-6.5, 5.1) |
| Togo | 10.9(9.1, 12.7) | 11.0(9.1, 12.9) | -0.1(-2.8, 2.5) |
| Tunisia | 13.5(10.4, 16.6) | 13.3(9.9, 16.6) | 0.2(-4.3, 4.8) |
| Turkmenistan | 10.7(8.4, 13.1) | 11.0(8.5, 13.5) | -0.3(-3.7, 3.2) |
| Uganda | 21.5(19.9, 23.2) | 23.2(21.5, 24.9) | -1.7(-4.1, 0.7) |
| Ukraine | 23.9(20.4, 27.4) | 25.6(21.7, 29.5) | -1.7(-7.0, 3.6) |
| Uruguay | 43.7(32.4, 54.9) | 31.7(21.6, 41.8) | 12.0(-3.2, 27.1) |
| Vietnam | 26.9(22.8, 31.0) | 25.9(21.9, 29.9) | 1.0(-4.8, 6.8) |
| Zimbabwe | 17.2(15.5, 19.0) | 18.4(16.6, 20.2) | -1.2(-3.7, 1.3) |
