# Supplementary material for: Maternal, paternal, and other caregivers’ stimulation in low- and- middle-income countries
Source: PLoS One. 2020 Jul 10;15(7):e0236107. doi: 10.1371/journal.pone.0236107 (PMC7351158; doi:10.1371/journal.pone.0236107)
Supplement: S12 Table — (DOCX) [file pone.0236107.s012.docx]

**S12 Table**. Area disparities in the percentage of children exposed to high maternal stimulation

| Country | Urban | Rural | Difference (Urban - Rural) |
| --- | --- | --- | --- |
| Afghanistan | 4.0(3.4, 4.6) | 7.3(5.9, 8.8) | 3.3(1.7, 4.9) |
| Algeria | 31.5(28.6, 34.3) | 43.1(41.0, 45.3) | 11.7(8.1, 15.2) |
| Argentina | 70.2(67.8, 72.7) |  |  |
| Bangladesh | 38.3(36.9, 39.7) | 50.8(47.1, 54.5) | 12.5(8.5, 16.5) |
| Belarus | 84.7(79.5, 89.9) | 85.5(82.7, 88.3) | 0.8(-5.1, 6.8) |
| Belize | 66.9(62.5, 71.3) | 72.2(66.9, 77.6) | 5.3(-1.6, 12.3) |
| Benin | 11.8(10.2, 13.3) | 15.8(14.0, 17.5) | 4.0(1.7, 6.3) |
| Bosnia and Herzegovina | 84.1(80.8, 87.4) | 90.3(85.9, 94.7) | 6.2(0.7, 11.7) |
| Burundi | 13.8(13.0, 14.6) | 33.7(30.2, 37.2) | 19.9(16.3, 23.5) |
| Cameroon | 12.3(10.4, 14.1) | 15.5(13.3, 17.8) | 3.2(0.3, 6.2) |
| Central African Republic | 20.6(18.9, 22.4) | 24.2(21.1, 27.2) | 3.5(-0.0, 7.1) |
| Congo, Dem. Rep. | 3.7(3.0, 4.5) | 6.9(5.4, 8.4) | 3.2(1.5, 4.9) |
| Congo, Rep. | 20.5(18.6, 22.5) | 30.3(27.1, 33.5) | 9.8(6.0, 13.6) |
| Costa Rica | 46.3(39.7, 52.9) | 48.4(39.2, 57.6) | 2.1(-9.2, 13.4) |
| Dominican Republic | 25.0(22.5, 27.4) | 34.1(32.2, 36.0) | 9.1(6.0, 12.2) |
| East Timor | 19.1(17.2, 21.1) | 21.2(17.4, 25.1) | 2.1(-2.2, 6.5) |
| El Salvador | 33.0(29.9, 36.0) | 46.4(43.1, 49.8) | 13.5(9.0, 18.0) |
| Gambia | 6.3(5.3, 7.3) | 7.4(5.5, 9.2) | 1.1(-1.0, 3.2) |
| Ghana | 6.7(5.0, 8.4) | 17.3(14.0, 20.6) | 10.6(6.9, 14.3) |
| Guinea | 12.5(11.0, 14.0) | 19.2(16.4, 22.0) | 6.7(3.5, 9.9) |
| Guinea-Bissau | 0.9(0.6, 1.3) | 6.4(4.2, 8.5) | 5.4(3.3, 7.6) |
| Guyana | 55.3(52.1, 58.5) | 61.1(55.1, 67.1) | 5.8(-1.0, 12.6) |
| Iraq | 16.4(12.9, 19.9) | 27.0(24.7, 29.2) | 10.6(6.4, 14.8) |
| Ivory Coast | 8.3(7.0, 9.6) | 20.7(17.6, 23.9) | 12.4(9.0, 15.9) |
| Jamaica | 56.9(50.3, 63.5) | 64.3(57.6, 70.9) | 7.3(-2.1, 16.7) |
| Jordan | 63.8(60.2, 67.3) | 67.4(64.5, 70.2) | 3.6(-1.0, 8.1) |
| Kazakhstan | 39.8(35.2, 44.5) | 63.5(60.1, 66.8) | 23.6(17.9, 29.3) |
| Kosovo | 38.7(33.9, 43.5) | 52.2(45.6, 58.9) | 13.5(5.3, 21.7) |
| Lao PDR | 15.1(13.7, 16.4) | 32.8(29.8, 35.8) | 17.7(14.5, 21.0) |
| Kyrgyzstan | 25.1(22.2, 28.0) | 44.4(39.3, 49.5) | 19.4(13.5, 25.2) |
| Lebanon | 50.1(44.0, 56.3) | 54.7(50.0, 59.4) | 4.6(-3.2, 12.3) |
| Macedonia | 53.3(45.5, 61.1) | 67.7(61.7, 73.7) | 14.4(4.6, 24.3) |
| Malawi | 9.1(8.2, 10.0) | 15.3(11.6, 19.0) | 6.2(2.4, 10.0) |
| Maldives | 87.0(85.0, 89.1) | 86.6(80.4, 92.8) | -0.4(-7.0, 6.1) |
| Mali | 18.5(17.3, 19.7) | 21.9(19.4, 24.4) | 3.4(0.6, 6.2) |
| Mauritania | 17.0(15.3, 18.6) | 25.7(23.1, 28.3) | 8.7(5.7, 11.8) |
| Mexico | 53.1(48.2, 58.0) | 65.9(60.6, 71.3) | 12.9(5.6, 20.1) |
| Moldova | 62.0(56.5, 67.5) | 80.7(76.8, 84.7) | 18.7(11.9, 25.5) |
| Mongolia | 23.7(21.0, 26.4) | 32.4(29.7, 35.1) | 8.7(4.9, 12.5) |
| Montenegro | 90.0(86.0, 94.0) | 93.1(90.4, 95.7) | 3.1(-1.8, 7.9) |
| Nepal | 27.7(25.1, 30.3) | 49.8(43.8, 55.8) | 22.2(15.6, 28.7) |
| Nigeria | 22.3(21.3, 23.3) | 41.8(39.5, 44.1) | 19.5(17.0, 22.0) |
| Palestine | 55.3(51.9, 58.7) | 55.5(53.4, 57.5) | 0.2(-3.8, 4.1) |
| Panama | 40.9(36.8, 44.9) | 62.5(57.2, 67.8) | 21.6(14.9, 28.3) |
| Paraguay | 33.0(29.3, 36.7) | 51.1(46.9, 55.4) | 18.1(12.5, 23.7) |
| Rwanda | 10.2(8.9, 11.5) | 16.7(13.5, 20.0) | 6.5(3.0, 10.0) |
| Senegal | 3.5(2.7, 4.2) | 10.9(8.7, 13.1) | 7.4(5.1, 9.7) |
| Serbia | 85.7(81.7, 89.7) | 91.7(89.3, 94.1) | 6.0(1.4, 10.7) |
| Sierra Leone | 12.6(11.5, 13.8) | 25.9(23.1, 28.6) | 13.2(10.2, 16.2) |
| St. Lucia | 70.5(60.2, 80.8) | 73.0(59.8, 86.1) | 2.5(-14.4, 19.4) |
| Suriname | 33.6(30.4, 36.7) | 44.5(39.5, 49.4) | 10.9(5.0, 16.8) |
| Swaziland | 12.8(10.4, 15.2) | 32.1(23.0, 41.3) | 19.3(9.9, 28.8) |
| São Tomé and Principe | 12.7(8.9, 16.6) | 17.6(13.7, 21.5) | 4.9(-0.6, 10.4) |
| Thailand | 57.2(53.6, 60.7) | 72.7(68.7, 76.7) | 15.6(10.2, 20.9) |
| Togo | 6.6(5.5, 7.7) | 11.7(9.3, 14.2) | 5.1(2.5, 7.8) |
| Tunisia | 28.7(23.8, 33.6) | 63.1(58.9, 67.3) | 34.4(27.9, 40.9) |
| Turkmenistan | 79.4(76.6, 82.2) | 87.2(84.4, 90.1) | 7.9(3.9, 11.9) |
| Uganda | 15.3(14.2, 16.5) | 30.2(26.9, 33.5) | 14.9(11.4, 18.4) |
| Ukraine | 87.3(84.1, 90.5) | 84.3(80.9, 87.6) | -3.0(-7.7, 1.6) |
| Uruguay | 62.6(44.9, 80.4) | 79.0(72.9, 85.0) | 16.3(-2.4, 35.1) |
| Vietnam | 38.7(34.8, 42.7) | 62.6(57.7, 67.5) | 23.9(17.6, 30.1) |
| Zimbabwe | 14.5(13.2, 15.9) | 27.5(24.5, 30.5) | 12.9(9.6, 16.2) |
