# Supplementary material for: Maternal, paternal, and other caregivers’ stimulation in low- and- middle-income countries
Source: PLoS One. 2020 Jul 10;15(7):e0236107. doi: 10.1371/journal.pone.0236107 (PMC7351158; doi:10.1371/journal.pone.0236107)
Supplement: S13 Table — (DOCX) [file pone.0236107.s013.docx]

**S13 Table**. Area disparities in the percentage of children exposed to high paternal stimulation

| Country | Urban | Rural | Difference (Urban - Rural) |
| --- | --- | --- | --- |
| Afghanistan | 3.9(3.3, 4.6) | 4.0(3.0, 5.0) | 0.0(-1.2, 1.2) |
| Algeria | 15.7(13.3, 18.0) | 18.4(16.6, 20.1) | 2.7(-0.2, 5.6) |
| Argentina | 22.3(20.1, 24.5) |  |  |
| Bangladesh | 8.7(7.9, 9.5) | 15.6(12.7, 18.5) | 6.9(3.8, 9.9) |
| Belarus | 26.8(20.1, 33.5) | 29.3(25.7, 32.9) | 2.5(-5.0, 10.1) |
| Belize | 23.6(19.9, 27.4) | 24.5(19.4, 29.6) | 0.8(-5.5, 7.2) |
| Benin | 4.2(3.2, 5.1) | 6.6(5.5, 7.8) | 2.5(1.0, 4.0) |
| Bosnia and Herzegovina | 26.0(22.1, 29.9) | 40.0(33.6, 46.4) | 14.0(6.5, 21.6) |
| Burundi | 2.4(2.0, 2.8) | 4.6(3.5, 5.8) | 2.2(1.0, 3.5) |
| Cameroon | 2.9(1.9, 3.9) | 5.0(3.5, 6.4) | 2.1(0.3, 3.8) |
| Central African Republic | 8.5(7.3, 9.8) | 8.7(6.7, 10.8) | 0.2(-2.2, 2.6) |
| Congo, Dem. Rep. | 1.5(0.9, 2.1) | 1.1(0.6, 1.7) | -0.4(-1.2, 0.5) |
| Congo, Rep. | 8.2(7.0, 9.5) | 5.2(3.7, 6.6) | -3.1(-5.0, -1.1) |
| Costa Rica | 12.8(8.3, 17.3) | 10.9(6.3, 15.5) | -1.9(-8.3, 4.5) |
| Dominican Republic | 5.9(4.5, 7.3) | 6.8(5.8, 7.9) | 1.0(-0.7, 2.7) |
| East Timor | 5.0(3.9, 6.0) | 4.6(3.0, 6.2) | -0.3(-2.2, 1.6) |
| El Salvador | 5.6(4.1, 7.0) | 9.9(8.0, 11.8) | 4.3(1.9, 6.7) |
| Gambia | 0.3(0.1, 0.5) | 1.7(0.7, 2.6) | 1.4(0.4, 2.4) |
| Ghana | 1.9(1.1, 2.8) | 5.3(3.4, 7.2) | 3.4(1.3, 5.4) |
| Guinea | 2.8(2.1, 3.5) | 7.3(5.5, 9.1) | 4.5(2.6, 6.5) |
| Guinea-Bissau | 0.1(0.0, 0.2) | 0.7(0.1, 1.3) | 0.5(-0.1, 1.1) |
| Guyana | 15.3(13.0, 17.6) | 19.2(14.2, 24.2) | 3.9(-1.6, 9.5) |
| Iraq | 6.5(5.0, 7.9) | 12.1(10.1, 14.0) | 5.6(3.2, 8.0) |
| Ivory Coast | 3.7(2.9, 4.4) | 7.1(5.1, 9.0) | 3.4(1.3, 5.5) |
| Jamaica | 10.5(6.2, 14.7) | 16.0(11.3, 20.7) | 5.5(-0.9, 11.8) |
| Jordan | 19.8(17.0, 22.7) | 20.9(18.3, 23.4) | 1.0(-2.8, 4.9) |
| Kazakhstan | 5.0(2.2, 7.8) | 8.5(6.7, 10.4) | 3.5(0.2, 6.9) |
| Kosovo | 7.5(4.7, 10.4) | 3.7(1.2, 6.3) | -3.8(-7.6, 0.0) |
| Lao PDR | 8.8(7.7, 9.9) | 16.6(14.2, 19.0) | 7.8(5.2, 10.4) |
| Kyrgyzstan | 2.3(1.3, 3.4) | 4.3(2.2, 6.3) | 1.9(-0.4, 4.2) |
| Lebanon | 9.1(5.5, 12.6) | 9.4(6.6, 12.1) | 0.3(-4.2, 4.8) |
| Macedonia | 13.9(7.7, 20.2) | 23.9(18.6, 29.2) | 10.0(1.7, 18.2) |
| Malawi | 2.8(2.3, 3.4) | 5.1(2.9, 7.3) | 2.2(0.0, 4.5) |
| Maldives | 17.4(15.1, 19.7) | 34.2(25.3, 43.2) | 16.8(7.6, 26.1) |
| Mali | 4.6(3.9, 5.3) | 7.3(5.7, 9.0) | 2.7(0.9, 4.5) |
| Mauritania | 3.0(2.3, 3.8) | 7.1(5.6, 8.6) | 4.0(2.3, 5.7) |
| Mexico | 11.6(8.3, 14.9) | 15.6(11.9, 19.3) | 4.0(-1.0, 9.0) |
| Moldova | 10.9(7.3, 14.5) | 17.1(13.5, 20.7) | 6.2(1.1, 11.3) |
| Mongolia | 8.7(6.9, 10.4) | 10.7(9.0, 12.5) | 2.1(-0.4, 4.6) |
| Montenegro | 35.4(28.4, 42.4) | 51.1(44.9, 57.3) | 15.7(6.3, 25.1) |
| Nepal | 9.1(7.5, 10.7) | 16.7(12.0, 21.4) | 7.6(2.6, 12.5) |
| Nigeria | 8.5(7.9, 9.2) | 16.3(14.6, 18.0) | 7.8(6.0, 9.6) |
| Palestine | 14.2(11.9, 16.5) | 11.6(10.3, 13.0) | -2.6(-5.3, 0.1) |
| Panama | 7.9(5.7, 10.1) | 18.0(13.6, 22.5) | 10.1(5.1, 15.1) |
| Paraguay | 10.6(8.0, 13.2) | 21.0(17.4, 24.6) | 10.4(5.9, 14.8) |
| Rwanda | 1.9(1.3, 2.5) | 3.2(1.8, 4.7) | 1.3(-0.2, 2.9) |
| Senegal | 0.5(0.2, 0.7) | 1.4(0.6, 2.2) | 1.0(0.1, 1.8) |
| Serbia | 25.4(20.5, 30.2) | 42.6(35.2, 49.9) | 17.2(8.4, 26.0) |
| Sierra Leone | 5.7(4.9, 6.5) | 10.1(8.2, 12.0) | 4.4(2.3, 6.5) |
| St. Lucia | 23.0(13.6, 32.4) | 18.5(6.8, 30.2) | -4.5(-19.7, 10.7) |
| Suriname | 4.9(3.4, 6.3) | 9.7(6.7, 12.7) | 4.8(1.5, 8.2) |
| Swaziland | 1.0(0.3, 1.7) | 5.1(1.3, 8.8) | 4.0(0.3, 7.8) |
| São Tomé and Principe | 2.9(1.1, 4.6) | 3.1(1.4, 4.8) | 0.3(-2.2, 2.7) |
| Thailand | 32.3(29.0, 35.7) | 37.4(32.5, 42.3) | 5.1(-0.8, 11.0) |
| Togo | 2.4(1.8, 3.1) | 3.2(1.8, 4.5) | 0.7(-0.8, 2.3) |
| Tunisia | 11.3(7.8, 14.9) | 24.7(20.8, 28.6) | 13.4(8.1, 18.7) |
| Turkmenistan | 14.6(12.1, 17.1) | 16.1(13.2, 19.0) | 1.5(-2.3, 5.3) |
| Uganda | 2.2(1.7, 2.6) | 4.9(3.2, 6.7) | 2.8(1.0, 4.6) |
| Ukraine | 24.8(20.3, 29.3) | 27.7(24.0, 31.3) | 2.8(-3.0, 8.6) |
| Uruguay | 11.8(5.5, 18.1) | 31.5(23.7, 39.3) | 19.7(9.7, 29.8) |
| Vietnam | 11.9(9.2, 14.6) | 23.0(18.6, 27.3) | 11.0(5.9, 16.2) |
| Zimbabwe | 1.8(1.3, 2.3) | 5.2(3.8, 6.6) | 3.4(1.9, 4.9) |
