# Supplementary material for: Maternal, paternal, and other caregivers’ stimulation in low- and- middle-income countries
Source: PLoS One. 2020 Jul 10;15(7):e0236107. doi: 10.1371/journal.pone.0236107 (PMC7351158; doi:10.1371/journal.pone.0236107)
Supplement: S14 Table — (DOCX) [file pone.0236107.s014.docx]

**S14 Table**. Area disparities in the percentage of children exposed to high stimulation by other caregivers

| Country | Urban | Rural | Difference (Urban - Rural) |
| --- | --- | --- | --- |
| Afghanistan | 13.3(12.2, 14.3) | 23.1(20.8, 25.5) | 9.9(7.3, 12.5) |
| Algeria | 21.1(18.6, 23.6) | 23.6(21.8, 25.4) | 2.5(-0.6, 5.6) |
| Argentina | 17.6(15.6, 19.6) |  |  |
| Bangladesh | 25.1(23.9, 26.3) | 25.3(22.1, 28.5) | 0.2(-3.2, 3.6) |
| Belarus | 18.2(12.3, 24.1) | 13.7(10.9, 16.5) | -4.6(-11.1, 2.0) |
| Belize | 25.7(21.6, 29.7) | 25.1(20.1, 30.1) | -0.6(-7.0, 5.8) |
| Benin | 4.4(3.4, 5.3) | 9.2(7.6, 10.8) | 4.9(3.0, 6.7) |
| Bosnia and Herzegovina | 36.6(31.9, 41.2) | 24.5(18.3, 30.6) | -12.1(-19.8, -4.4) |
| Burundi | 29.9(28.9, 30.9) | 37.8(34.3, 41.2) | 7.9(4.3, 11.5) |
| Cameroon | 24.3(21.8, 26.7) | 24.2(21.5, 26.9) | -0.0(-3.7, 3.7) |
| Central African Republic | 38.7(36.3, 41.0) | 27.8(24.8, 30.8) | -10.8(-14.6, -7.0) |
| Congo, Dem. Rep. | 11.2(9.9, 12.5) | 13.3(11.4, 15.2) | 2.2(-0.1, 4.5) |
| Congo, Rep. | 14.7(13.1, 16.2) | 23.1(20.2, 26.0) | 8.4(5.1, 11.7) |
| Costa Rica | 14.5(9.2, 19.7) | 17.1(10.3, 23.9) | 2.6(-6.0, 11.2) |
| Cote d'Ivoire | 5.8(4.8, 6.8) | 13.4(10.8, 16.0) | 7.6(4.8, 10.4) |
| Dominican Republic | 15.4(13.7, 17.2) | 16.8(15.2, 18.4) | 1.4(-1.0, 3.7) |
| East Timor | 3.7(2.8, 4.6) | 6.5(4.1, 9.0) | 2.8(0.2, 5.4) |
| El Salvador | 5.9(4.6, 7.3) | 7.4(5.6, 9.2) | 1.4(-0.8, 3.7) |
| Gambia | 39.0(36.7, 41.4) | 32.8(29.4, 36.2) | -6.3(-10.4, -2.1) |
| Ghana | 14.6(12.4, 16.8) | 24.0(20.0, 27.9) | 9.4(4.8, 13.9) |
| Guinea | 10.0(8.7, 11.4) | 20.8(17.9, 23.6) | 10.8(7.6, 13.9) |
| Guinea-Bissau | 17.2(15.4, 18.9) | 19.7(16.4, 23.0) | 2.5(-1.2, 6.3) |
| Guyana | 32.9(29.9, 35.9) | 35.3(29.3, 41.2) | 2.4(-4.3, 9.1) |
| Iraq | 15.5(11.1, 19.9) | 15.2(13.4, 17.0) | -0.3(-5.0, 4.5) |
| Ivory Coast | 5.8(4.8, 6.8) | 13.4(10.8, 16.0) | 7.6(4.8, 10.4) |
| Jamaica | 39.9(33.4, 46.4) | 36.9(30.6, 43.2) | -3.0(-12.0, 6.1) |
| Jordan | 8.9(6.9, 11.0) | 7.6(6.0, 9.2) | -1.3(-3.9, 1.3) |
| Kazakhstan | 32.2(27.6, 36.8) | 23.1(20.2, 25.9) | -9.2(-14.6, -3.7) |
| Kosovo | 16.2(12.6, 19.8) | 12.5(8.2, 16.9) | -3.6(-9.3, 2.0) |
| Lao PDR | 13.3(12.0, 14.7) | 20.1(17.5, 22.6) | 6.7(3.9, 9.6) |
| Kyrgyzstan | 26.1(23.1, 29.0) | 19.7(15.7, 23.7) | -6.4(-11.3, -1.4) |
| Lebanon | 19.5(14.6, 24.4) | 17.5(13.9, 21.2) | -2.0(-8.1, 4.1) |
| Macedonia | 18.9(13.5, 24.3) | 22.5(17.3, 27.6) | 3.6(-3.9, 11.1) |
| Malawi | 10.1(9.1, 11.0) | 12.7(9.5, 15.9) | 2.6(-0.7, 5.9) |
| Maldives | 18.1(15.8, 20.5) | 26.2(17.9, 34.5) | 8.1(-0.6, 16.7) |
| Mali | 23.7(22.4, 25.1) | 25.1(22.3, 27.8) | 1.3(-1.7, 4.4) |
| Mauritania | 15.6(14.1, 17.2) | 22.1(19.7, 24.5) | 6.5(3.6, 9.3) |
| Mexico | 11.2(7.8, 14.5) | 11.3(8.2, 14.4) | 0.1(-4.4, 4.7) |
| Moldova | 25.4(20.5, 30.4) | 18.9(15.0, 22.8) | -6.5(-12.8, -0.2) |
| Mongolia | 9.5(7.6, 11.4) | 17.8(15.6, 19.9) | 8.2(5.3, 11.1) |
| Montenegro | 39.6(32.4, 46.8) | 27.3(21.9, 32.7) | -12.3(-21.4, -3.3) |
| Nepal | 30.4(27.7, 33.1) | 24.8(19.7, 29.9) | -5.6(-11.4, 0.1) |
| Nigeria | 29.9(28.7, 31.1) | 33.8(31.6, 36.0) | 3.9(1.4, 6.4) |
| Palestine | 15.0(12.6, 17.5) | 14.9(13.5, 16.4) | -0.1(-3.0, 2.7) |
| Panama | 16.2(13.1, 19.2) | 15.7(11.7, 19.7) | -0.5(-5.5, 4.6) |
| Paraguay | 12.5(9.8, 15.2) | 21.2(17.6, 24.8) | 8.7(4.2, 13.1) |
| Rwanda | 20.3(18.6, 22.0) | 19.6(16.0, 23.2) | -0.7(-4.6, 3.3) |
| Senegal | 11.5(10.3, 12.8) | 15.0(12.5, 17.5) | 3.5(0.7, 6.3) |
| Serbia | 25.8(19.5, 32.1) | 14.2(9.9, 18.5) | -11.6(-19.2, -4.0) |
| Sierra Leone | 2.2(1.7, 2.8) | 4.8(3.5, 6.2) | 2.6(1.1, 4.0) |
| St. Lucia | 43.2(32.1, 54.4) | 31.2(17.5, 44.9) | -12.0(-29.9, 5.9) |
| Suriname | 21.8(19.1, 24.6) | 24.6(20.3, 29.0) | 2.8(-2.3, 8.0) |
| Swaziland | 18.2(15.6, 20.8) | 11.9(5.9, 17.9) | -6.3(-12.8, 0.2) |
| São Tomé and Principe | 34.4(29.1, 39.8) | 27.5(23.1, 32.0) | -6.9(-13.9, 0.1) |
| Thailand | 51.3(47.7, 54.8) | 54.7(49.8, 59.6) | 3.4(-2.6, 9.5) |
| Togo | 12.0(10.4, 13.6) | 9.2(6.9, 11.5) | -2.8(-5.6, -0.0) |
| Tunisia | 13.0(9.3, 16.7) | 13.6(10.7, 16.5) | 0.6(-4.1, 5.3) |
| Turkmenistan | 10.5(8.3, 12.7) | 11.6(8.8, 14.3) | 1.1(-2.5, 4.6) |
| Uganda | 21.9(20.6, 23.1) | 24.4(21.5, 27.3) | 2.6(-0.6, 5.7) |
| Ukraine | 28.1(23.5, 32.6) | 23.3(20.1, 26.5) | -4.7(-10.3, 0.9) |
| Uruguay | 29.9(12.2, 47.6) | 39.0(30.5, 47.4) | 9.1(-10.6, 28.7) |
| Vietnam | 27.4(23.7, 31.0) | 24.0(19.6, 28.3) | -3.4(-9.1, 2.3) |
| Zimbabwe | 18.5(17.1, 20.0) | 15.6(13.4, 17.9) | -2.9(-5.6, -0.2) |
